# B1 SOX Coordinate Cell Specification with Patterning and Morphogenesis in the Early Zebrafish Embryo

**DOI:** 10.1371/journal.pgen.1000936

**Published:** 2010-05-06

**Authors:** Yuichi Okuda, Eri Ogura, Hisato Kondoh, Yusuke Kamachi

**Affiliations:** Graduate School of Frontier Biosciences, Osaka University, Suita, Japan; Medical Research Council Human Genetics Unit, United Kingdom

## Abstract

The B1 SOX transcription factors SOX1/2/3/19 have been implicated in various processes of early embryogenesis. However, their regulatory functions in stages from the blastula to early neurula remain largely unknown, primarily because loss-of-function studies have not been informative to date. In our present study, we systematically knocked down the B1 *sox* genes in zebrafish. Only the quadruple knockdown of the four B1 *sox* genes *sox2/3/19a/19b* resulted in very severe developmental abnormalities, confirming that the B1 *sox* genes are functionally redundant. We characterized the *sox2/3/19a/19b* quadruple knockdown embryos in detail by examining the changes in gene expression through in situ hybridization, RT–PCR, and microarray analyses. Importantly, these phenotypic analyses revealed that the B1 SOX proteins regulate the following distinct processes: (1) early dorsoventral patterning by controlling *bmp2b/7*; (2) gastrulation movements via the regulation of *pcdh18a/18b* and *wnt11*, a non-canonical Wnt ligand gene; (3) neural differentiation by regulating the *Hes*-class bHLH gene *her3* and the proneural-class bHLH genes *neurog1* (positively) and *ascl1a* (negatively), and regional transcription factor genes, e.g., *hesx1*, *zic1*, and *rx3*; and (4) neural patterning by regulating signaling pathway genes, *cyp26a1* in RA signaling, *oep* in Nodal signaling, *shh*, and *mdkb*. Chromatin immunoprecipitation analysis of the *her3*, *hesx1*, *neurog1*, *pcdh18a*, and *cyp26a1* genes further suggests a direct regulation of these genes by B1 SOX. We also found an interesting overlap between the early phenotypes of the B1 *sox* quadruple knockdown embryos and the maternal-zygotic *spg* embryos that are devoid of *pou5f1* activity. These findings indicate that the B1 SOX proteins control a wide range of developmental regulators in the early embryo through partnering in part with Pou5f1 and possibly with other factors, and suggest that the B1 *sox* functions are central to coordinating cell fate specification with patterning and morphogenetic processes occurring in the early embryo.

## Author Summary

In the developing embryo, various processes such as cell fate specification, embryo patterning, and morphogenesis take place concurrently. The embryo must control gene expression in order to coordinate these processes and thereby enable the proper organization of its structures. The B1 *sox* transcription factor genes, exemplified by the “stem cell gene” *sox2*, are thought to play a key role in these embryonic processes from the blastoderm stage to the neural stage. However, the precise regulatory functions of these genes are largely unknown due to the lack of loss-of-function studies. In our current study, we took advantage of the zebrafish system and successfully depleted B1 *sox* activity from the early embryo using antisense knockdown technology. This approach enabled us to further uncover the regulatory functions of B1 *sox* in early embryos. We found that the activity of the B1 *sox* genes is required for the expression of a wide range of developmental regulators including transcription factors, signaling pathway components, and cell adhesion molecules. These findings suggest that the B1 *sox* functions are central to coordinating diverse embryonic processes, particularly those that occur during the development of the primordium of the central nervous system.

## Introduction

The developing embryo must control gene expression to coordinate various embryonic processes such as cell fate specification, embryo patterning and morphogenesis. During the embryonic stages from the blastula to neurula, the coupling of cell lineage specification and gastrulation cell movements is particularly evident. There is also now an increased understanding of the regulatory mechanisms underlying each cell state and each morphogenetic process, but the precise mechanisms that coordinate these events have remained elusive. The group B1 SOX transcription factors are good candidates as coordinators of these embryonic processes. Indeed, they have been implicated in cell fate specification in the early embryo [1]–[7] and also patterning and morphogenetic processes [8]–[10].

B1 *Sox* comprises *sox1a/1b*/*2*/*3*/*19a*/*19b* in zebrafish and *Sox1*/*2*/*3* in amniotes [11]. The *sox19a/19b* genes are evolutionary orthologs of mammalian *Sox15* (group G), although *Sox15* has now been shown to have functionally diversified from the authentic B1 *Sox* paralogs [11]. Overall, the regulatory functions of B1 *sox* genes appear to be conserved as a group across vertebrate species, although the paralogs are often differentially employed in a particular process [12]. In zebrafish, *sox3/19a/19b* are expressed in the blastula [11], whereas the corresponding early expression in mice is covered by *Sox2* [1]. Following this stage, the B1 *sox* genes are thought to be important for specification of the embryonic ectoderm into the neuroectoderm lineage. During this process, their expression becomes confined to the neuroectoderm [11]. As development proceeds to the neurula stage, expression of the B1 *sox* genes continues in neural precursors, where they function to maintain the neural progenitor states [13]–[15].

The similarities in the characteristics of the B1 SOX proteins as transcriptional regulators [11], [15] suggest redundant functions in tissues where they are coexpressed. In support of this notion, single *Sox1* or *Sox3* knockout mice display only mild abnormalities in the central nervous system (CNS), presumably because of extensive coexpression of *Sox1/2/3* [16]–[18], whereas *Sox2*-null mouse embryos die around implantation, reflecting its exclusive expression in the ICM [1]. Consistently, a single *sox2* or *sox3* knockdown (KD) in zebrafish causes only mild developmental abnormalities [19], [20]. *Xenopus* studies utilizing dominant-negative forms of SOX2 indicate a specific role of *Sox2* in neuroectoderm differentiation [2]. To date, however, the overall functions of the B1 *sox* genes have not been systematically investigated from the blastula to early neurula stages.

An important characteristic of the B1 SOX proteins is that they form a complex with co-DNA-binding partner factors to target specific sequences and this enables them to participate in the regulation of various cell states [21]. The SOX2-Oct3/4 (Pou5f1) complex is a central player in regulatory networks in the ICM and ES cells [1], [22], [23]. Potential target genes of SOX2 and Oct3/4 in ES cells have been identified through genome-wide chromatin immunoprecipitation (ChIP) and microarray expression analyses [22], [24]. The involvement of other B1 SOX-partner combinations in the regulation of specific cell states has also been reported, e.g., B1 SOX-POUIII factors in neural precursors [15] and B1 SOX-Pax6 in lens cells [25]. However, neither B1 SOX-dependent regulatory processes nor B1 SOX target genes in the developing early embryo have been extensively investigated.

In our present study, we performed single to quadruple knockdowns of *sox2/3/19a/19b* in zebrafish embryos and confirmed that these four genes are functionally redundant in early development. More importantly, phenotypic analyses of the *sox2/3/19a/19b* quadruple KD embryos uncovered developmental process-specific functions of B1 *sox*. In the blastula, B1 *sox* genes regulate the activation of the *bmp2b/7* genes, which is critical for dorsoventral (DV) patterning. During gastrulation, B1 *sox* also regulate the expression of *pcdh18a/18b* and *wnt11*, a non-canonical Wnt ligand gene, which together play a role in convergence and extension (C&E) movements. In neural development, the B1 *sox* genes are essential for the proper regulation of neural bHLH genes of both the *her/Hes* and proneural classes, and also for the activation of region-specific transcription factor genes such as *hesx1*, *zic1* and *rx3*. Moreover, the activity of B1 *sox* is required for the neural expression of various signaling pathway genes: *cyp26a1* in RA signaling, *oep* in Nodal signaling, *shh*, and also *mdkb*. ChIP analysis of the *her3*, *hesx1*, *neurog1*, *pcdh18a* and *cyp26a1* genes suggests their direct regulation by B1 SOX. These findings indicate that B1 SOX proteins play a central role in coordinating cell fate specification, embryo patterning and morphogenesis by controlling a wide variety of developmental regulators in the early embryo.

We have also found an interesting overlap between the early phenotypes of the B1 *sox* quadruple KD embryos and the maternal-zygotic (MZ) *spg* embryos that are devoid of *pou5f1* activity [26]–[29]. This highlights a broad role of the B1 SOX-Pou5f1 complex from the blastoderm to early neural stages of development.

## Results

### Loss-of-function analysis of the B1 *sox* genes in the zebrafish embryo

Among the B1 *sox* genes of zebrafish, *sox2/3/19a/19b* are expressed at high levels during early development with extensive regional overlaps [11]. *sox19b* mRNA is maternally supplied. *sox3* and *sox19a* are activated around the 1000-cell stage, and *sox2* around the 30% epiboly (30%E) stage [11]. The expression of *sox3/19a/19b* initially covers the entire blastoderm, but gradually disappears at the embryonic margin after 30%E (Figure S1A). At the shield stage, the expression of *sox2/3/19a/19b* covers the future ectoderm, but then becomes confined to the presumptive neuroectoderm [11]. Expression of *sox1a/1b* is initiated only during late gastrulation stages (Figure S1B). These expression patterns suggest that *sox2/3/19a/19b* are involved in early processes of zebrafish development.

To investigate the function of B1 *sox* in early stage embryos, we knocked down *sox2/3/19a/19b* either individually or in combination using morpholino antisense oligonucleotides (MO). Two different MOs were simultaneously used to block translation of each B1 *sox* gene, which ensures efficient knockdown even when using reduced amounts of MOs [20]. With this double MO strategy, an approximately 90% reduction in translation was achieved using 1.8 ng of a 1∶1 mixture of two MOs, as judged by their effects on luciferase reporters carrying MO-targeting 5′-UTR sequences (*sox2* [20]; *sox3/19a/19b*, Figure S2A). By western blotting, we confirmed the efficient inhibition of the synthesis of endogenous B1 SOX proteins (Figure S2B).

**Figure 1:**
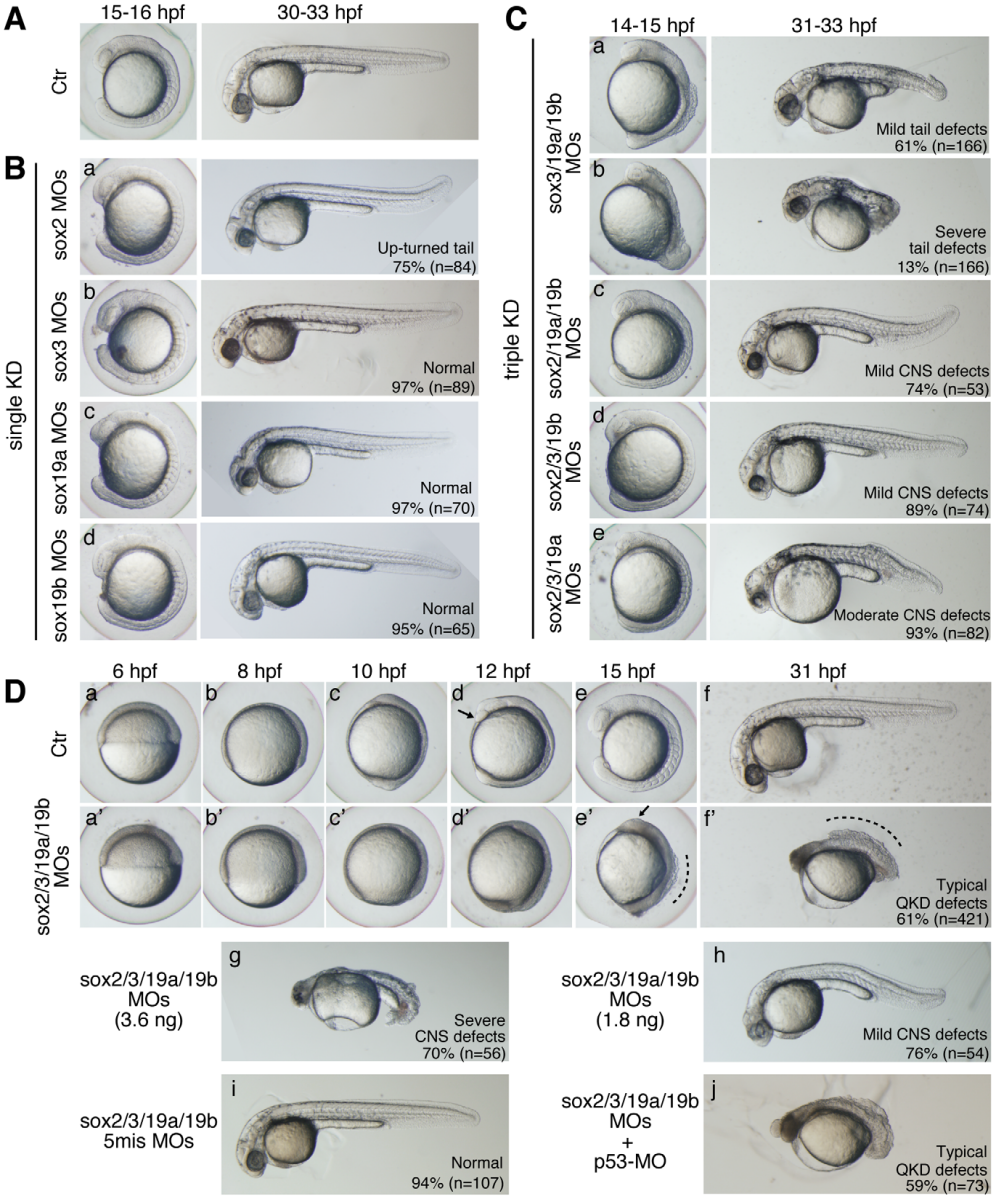
Single, triple, and quadruple knockdowns of *sox2/3/19a/19b*. Bright-field images of live embryos observed at the indicated time points. All are lateral views. (A) Uninjected control (Ctr) embryos. (B) Single knockdowns of *sox2/3/19a/19b*. A 1∶1 mixture of two MOs (0.9 ng each) targeting one of the four B1 *sox* genes was injected for a single KD. The percentage of embryos in the same morphological class is indicated in each panel. (C) Triple knockdowns of *sox2/3/19a/19b*. A mixture of indicated combinations of MOs (i.e., a mixture of six MOs, 5.4 ng in total) was used to simultaneously knockdown three out of the four B1 *sox* genes. The major classes of morphological defects are shown with the percentage of occurrence. The remaining embryos showed either milder or more severe defects. (D) Uninjected control embryos (a–f) and *sox2/3/19a/19b* quadruple knockdown (QKD) embryos injected with a mixture of MOs targeting the four B1 *sox* genes (i.e., a mixture of eight MOs, 7.2 ng in total) (a’–f’). The QKD caused very severe developmental abnormalities: a delay in epiboly, a shortened anterior-posterior axis, and impairment of CNS development (61%). The remaining embryos showed either milder defects (7%), more severe defects (24%) or lethality (8%). The aberrant movement of the anterior prechordal plate (arrows in d and e’) suggests a decreased adhesion of ectodermal cells as well as defects in convergence and extension movements in the QKD embryos. The broken lines (e’ and f’) indicate the dorsal trunk regions where cell dissociation was observed. (g, h) Dose-dependent effects of the MOs used for QKD were examined by injecting reduced amounts of the mixture of MOs targeting the four B1 *sox* genes (3.6 ng in total [g] and 1.8 ng in total [h]). (i) As a negative control, a mixture of 5-base-mismatch control MOs (i.e., a mixture of eight 5mis-MOs, 7.2 ng in total) was injected. (j) The coinjection of a p53-MO (2 ng) had no impact on the neural defects in the QKD embryos.

No gross abnormalities were observed in the embryo morphology when any one of *sox2/3/19a/19b* was knocked down (single KD, Figure 1B), although the development of the CNS may be slightly perturbed and 75% of the *sox2* morphants showed an up-turned tail phenotype (Figure 1Ba). When any three of *sox2/3/19a/19b* were simultaneously knocked down (triple KD), a range of morphological abnormalities was observed depending on the combination of KD targets (Figure 1C). Triple KDs of *sox2/19a/19b* and *sox2/3/19b* caused only mild morphological defects (Figure 1Cc and 1Cd), presumably because the remaining *sox3* and *sox19a* genes, respectively, mostly cover the B1 *sox* expression domains. *sox3/19a/19b* morphants often showed stronger yet variable defects in their posterior structures (Figure 1Ca and 1Cb), presumably reflecting the weak *sox2* expression in the posterior neuroectoderm. *sox2/3/19a* morphants appeared normal during gastrulation, but later developed morphological abnormalities (Figure 1Ce), likely because *sox19b* expression decreases in later stages.

In contrast to the triple KDs, the quadruple knockdown of *sox2/3/19a/19b* (hereafter called QKD) using a total of 7.2 ng MOs resulted in very severe developmental abnormalities, suggesting essential functions of B1 *sox* in early embryogenesis (Figure 1Da–1Df). This result also lends support to a model whereby in the triple KD embryos the remaining B1 *sox* gene compensates for the loss of the other three to a large extent. Taken together, our initial observations indicate that the B1 *sox* genes are largely functionally redundant in early zebrafish embryos. This is further corroborated by rescue experiments to be described in the next section.

By reducing the amount of MOs used for QKD, hypomorphic phenotypes were produced to different extents depending on the MO levels (Figure 1Dg and 1Dh). Embryos injected with a 50% concentration of MOs for QKD (3.6 ng in total) still showed more severe abnormalities than any of the triple KD embryos. However, injection of only 25% MOs for QKD (1.8 ng in total) resulted in phenotypes similar to the triple KD embryos in terms of severity, further demonstrating the dose-dependent knockdown effects.

The specificity of the MOs we used was confirmed by injection of 5-base mismatch MOs, which caused no developmental abnormalities (Figure 1Di). In addition, the observed QKD phenotypes were not altered by coinjection of p53-MO (Figure 1Dj), which relieves MO-induced non-specific neural cell death [30]. Efficient elimination of B1 SOX activity under this QKD condition was also confirmed by the loss of *nestin* enhancer activity (Figure S3), which is regulated by B1 SOX and POU [15].

**Figure 2:**
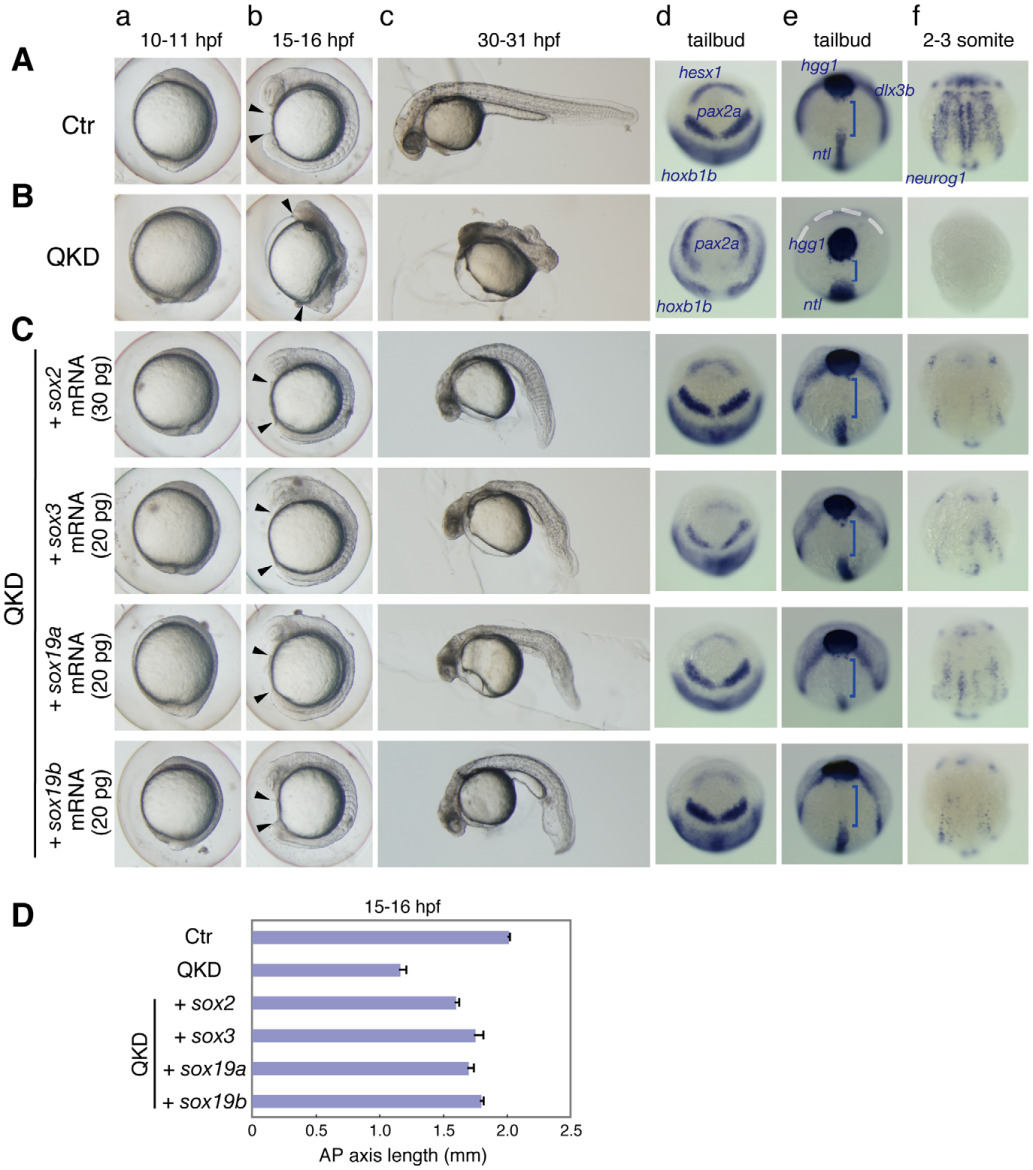
Rescue of the QKD phenotype by exogenous B1 *sox* mRNAs. (A, B) Uninjected control (Ctr) embryos (A) and the QKD embryos (B). Live embryos were observed at 10–11 (a), 15–16 (b) and 30–31 (c) hpf. Expression of *hesx1*, *pax2a* and *hoxb1b* (d), *dlx3b*, *hgg1* and *ntl* (e), and *neurog1* (f) was visualized by whole-mount in situ hybridization. Lateral views (a–c); dorsal views with anterior to the top (d–f). (C) The QKD phenotype is similarly rescued by an exogenous supply of any B1 *sox* mRNA. The MOs for QKD were coinjected with the indicated mRNAs. In the B1 *sox* mRNA-coinjected embryos, the expression of *hesx1*, *dlx3b* and *nuerog1* was recovered; patterning of the neural plate marked by *pax2a* and *hoxb1b* was normalized; and the expression patterns of *hgg1*, *ntl* and *dlx3b* reflecting C&E movements were also restored. Blue bracket, gap between the *hgg1* and *ntl* expression domains; white dotted line, neural plate border. (D) Recovery of the AP axis elongation in QKD embryos injected with one of the B1 *sox* mRNAs. The length of the embryos at 15–16 hpf along the AP axis between the arrowheads (Ab, Bb, Cb) was measured for the uninjected control (Ctr) (n = 7), the QKD (n = 9) and QKD with B1 *sox* mRNA injection (*sox2*, n = 9; *sox3*, n = 6; *sox19a*, n = 7; *sox19b*, n = 6). The average AP axis lengths with standard errors are shown.

**Figure 3:**
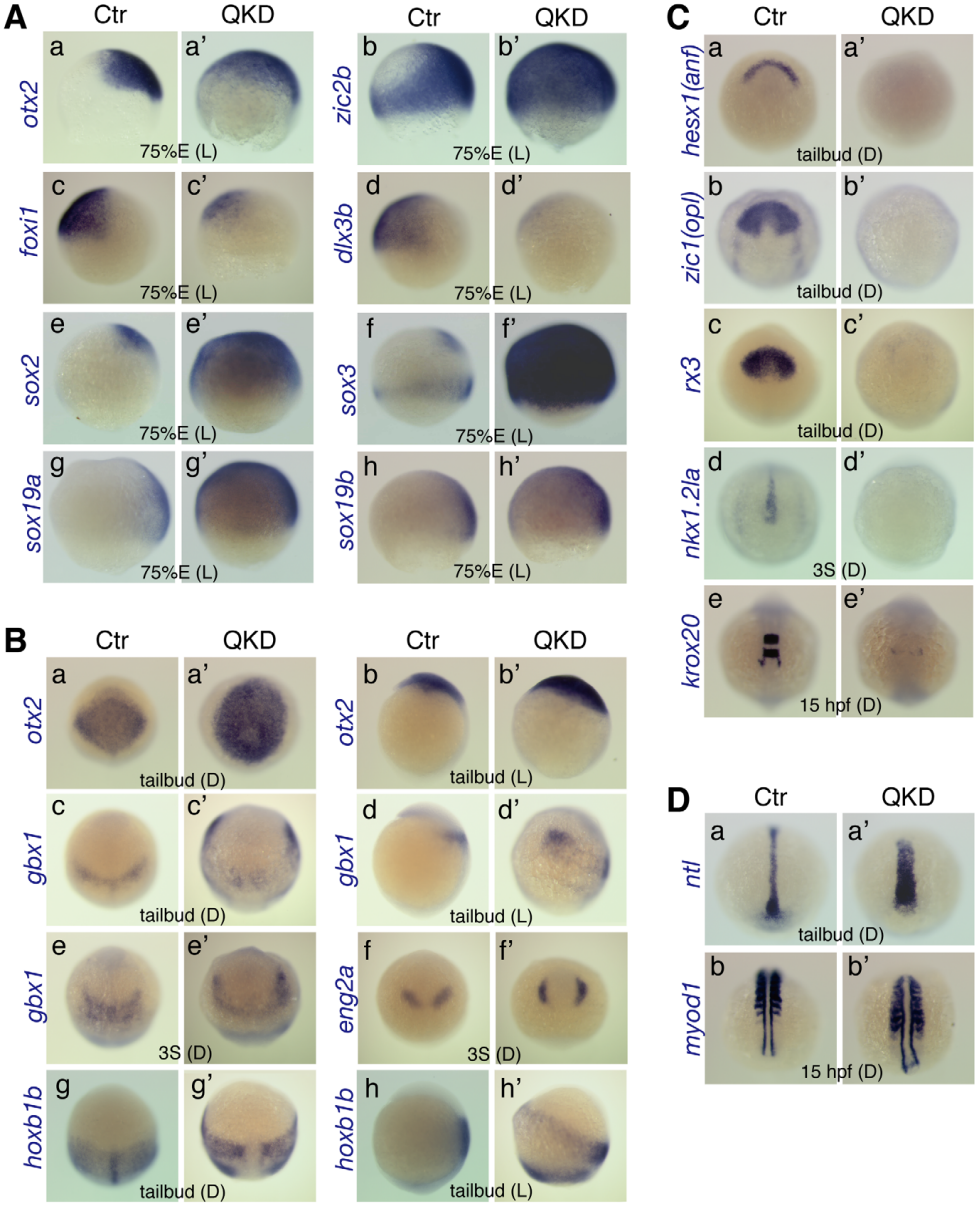
Defects in embryo patterning, early neural development, and C&E movements in the QKD embryos. Comparison of the gene expression profiles between uninjected control (Ctr) and QKD embryos. (A) Ventral expansion of early neural gene expression (*otx2* [a], *zic2b* [b] and *sox2/3/19a/19b* [e–h]), and reduced expression of non-neural ectoderm marker genes (*foxi1* [c] and *dlx3b* [d]) at 75%E. (B) Altered expression patterns of neural genes at the tailbud and 3-somite stages. The expression domains of *gbx1* (c–e), *eng2a* (f) and *hoxb1b* (g, h) were anterolaterally shifted, which encompass the *otx2* expression domain (a, b). (C) Defective neural development revealed by the loss of expression of neural marker genes, *hesx1* (a), *zic1* (b), *rx3* (c), *nkx1.2la* (d) and *krox20* (e). Residual expression of *krox20* was observed in r5-derived neural crest cells. (D) Wider mesoderm structures with a shortened axis revealed by mesodermal marker genes, *ntl* (a) and *myod1* (b). The stages and embryo orientations are shown in each panel: E, epiboly stage; S, somite stage; L, lateral view with dorsal to the right; D, dorsal view with anterior to the top.

The earliest detectable morphological abnormality of the QKD embryos was a delay in epiboly, notably after the shield stage (Figure 1D). At 10 hour post-fertilization (hpf), when normal embryos reach the tailbud stage, the QKD embryos were still in late epiboly. The thickening of the anterior head region was less prominent in the QKD embryos (Figure 1Dc and 1Dd), suggesting impairment of CNS development. Impaired CNS development was also indicated by the loss of *hesx1* expression in the anterior-most neuroectoderm (Figure 2Bd; see also Figure 3Ca) and by the anterolateral displacement of the *pax2a* expression domains that mark the midbrain-hindbrain boundary (MHB) (Figure 2Bd). An early phase of neurogenesis was also affected in the QKD embryos as indicated by the loss of proneural *neurog1* expression (Figure 2Bf). The QKD embryos further displayed a shortened anterior-posterior (AP) axis with a broadened neural plate (marked by *hoxb1b*) and broadened mesodermal structures including notochord (marked by *ntl*) (Figure 1D and Figure 2B). Consistently, the gap between the prechordal plate (marked by *hgg1*) and notochord was reduced in the QKD embryos (Figure 2Be). These abnormalities commonly occur in zebrafish embryos when C&E movements are impaired during gastrulation [31], [32].

### Interchangeable B1 SOX protein functions during early zebrafish development

The effects of the B1 *sox* KDs suggested that the B1 SOX proteins act equivalently in transcriptional regulation in the zebrafish embryo. Indeed, SOX1/2/3/19 all activate the *nestin* and *δ-crystallin* enhancers in cooperation with Brn2 and Pax6, respectively, in cultured cells [11]. We therefore tested whether injection of a single B1 *sox* mRNA could rescue the QKD phenotype. Moderate amounts (20–30 pg) of B1 *sox* mRNAs lacking the MO-target 5′ UTR sequences were individually injected with the MOs for QKD. Coinjection with any one of the *sox2/3/19a/19b* mRNAs dramatically rescued the QKD phenotype, as judged by the recovery of a normal morphology (Figure 2Ca–2Cc). By measuring the AP axis length of the embryos at 15–16 hpf, we confirmed the recovery of axial elongation in the B1 *sox* mRNA-injected QKD embryos (Figure 2D). In these embryos also, the expression of *hesx1*, *dlx3b* (neural plate border) and *neurog1* was recovered and the expression patterns of *pax2a* and *hoxb1b* were restored (Figure 2Cd–2Cf). In addition, the C&E movements indicated by the expression patterns of *hgg1*, *ntl* and *dlx3b* [31] were normalized in the B1 *sox* mRNA-injected embryos (Figure 2Ce). This phenotypic rescue was efficient only to early somitogenesis stages, likely because of the gradual decrease in the exogenously supplied SOX expression [19]. Simultaneous injection of *sox2/3/19a/19b* mRNAs (5 pg each) had essentially the same rescue effects (data not shown). These observations indicate that the function of the B1 SOX proteins is interchangeable during early zebrafish development.

### Phenotypes of B1 *sox*-deficient embryos

To further explore the functions of B1 *sox* in early zebrafish embryogenesis, we characterized our QKD embryos by focusing on their defects in neural development and gastrulation movements. In these QKD embryos, expression domains of *otx2* (anterior neuroectoderm) and *zic2b* (entire neuroectoderm) were expanded ventrally at 75%E, whereas expression of both *foxi1* and *dlx3b* (non-neuroectoderm) was reduced (Figure 3Aa–3Ad). Consistently, the neuroectodermal expression of B1 *sox* was also expanded in the QKD embryo (Figure 3Ae–3Ah). These gene expression changes are reminiscent of the dorsalized phenotype seen in BMP-pathway mutants [33], [34], suggesting impairment of this pathway in the QKD embryos.

At the tailbud stage in the QKD embryos, expression domains of *pax2a*, *gbx1* (hindbrain) and *hoxb1b* (posterior neuroectoderm) were anterolaterally shifted, which encompass the *otx2* expression domain (Figure 2Bd and Figure 3B). A similar change was seen for *eng2a* expression in the MHB at the 3-somite stage (Figure 3Bf). These observations indicate that some characteristics of the early neural plate can develop even under a severe reduction of B1 SOX activity. However, our initial analyses revealed that expression of many neural genes is abolished in the QKD embryos, including *zic1* (forebrain), *rx3* (eye field), *nkx1.2la* (posterior neuroectoderm) and *krox20* (rhombomere [r] 3/5) as well as *hesx1* and *neurog1* (Figure 2B and Figure 3C). Injection of the MOs for QKD into embryos of the *nr2f2* enhancer-trap line [35] confirmed the impairment of brain development at later stages (Figure S4A). These findings together suggest that B1 *sox* activity is critical for neural development, although it is dispensable for the expression of some early neural genes.

The normal expression levels of *ntl* and *myod1* (somite) in the QKD embryos suggest that mesodermal differentiation per se can proceed (Figure 3D). However, the broadened expression domains of these genes indicate defects in convergence movements, which is consistent with the widened expression of neural genes such as *gbx1* and *hoxb1b*. Movement of the anterior prechordal plate (marked by *hgg1*) was also impaired in the QKD embryos (Figure 2Be), which is characteristic of defective C&E movements. However, hatching gland precursor cells were commonly found to aberrantly move in a dorsal direction and penetrated the ectoderm during mid-somite stages (Figure 1De’). In the severe morphants, these hatching gland cells remained as a single ball-like structure in the head (Figure S4B). This phenotype is unique to the QKD embryo and may reflect a decreased adhesion of ectodermal cells, as also suggested by cell dissociation from the dorsal trunk region (Figure 1De’ and 1Df’).

Interestingly, the QKD embryos show an increase in transcript levels of *sox2/3/19a/19b* and also *sox1b* at early developmental stages (Figure 3A and Figure S1B). This implies a negative autoregulation of transcription among the B1 *sox* members, although a stabilization of these mRNAs by the MOs could not be ruled out. It is also noteworthy that even with these elevated levels of B1 *sox* transcripts in the QKD embryos, the MO-mediated knockdowns were effective in inhibiting their translation as revealed by western blotting (Figure S2B).

To further characterize the phenotype of the QKD embryos, we examined the changes in gene expression in greater detail by the combined use of in situ hybridization, RT-PCR (summarized in Table S1) and microarray analysis (Figure S5, Table S2 and ). Overall, these analyses indicated that a wide range of developmental processes were affected in the QKD embryos and the major phenotypes can be categorized into: (1) the early dorsoventral patterning defects; (2) defects in gastrulation movements; (3) dysregulation of early neural and neuronal regulatory genes; (4) neural patterning defects associated with the abrogated expression of signaling pathway genes; and (5) early defects resembling those observed for MZ*spg* embryos. Further details of these phenotypes are described below.

### Aberrant DV patterning in B1 *sox* QKD embryos caused by dysregulation of *bmp* genes

**Figure 4:**
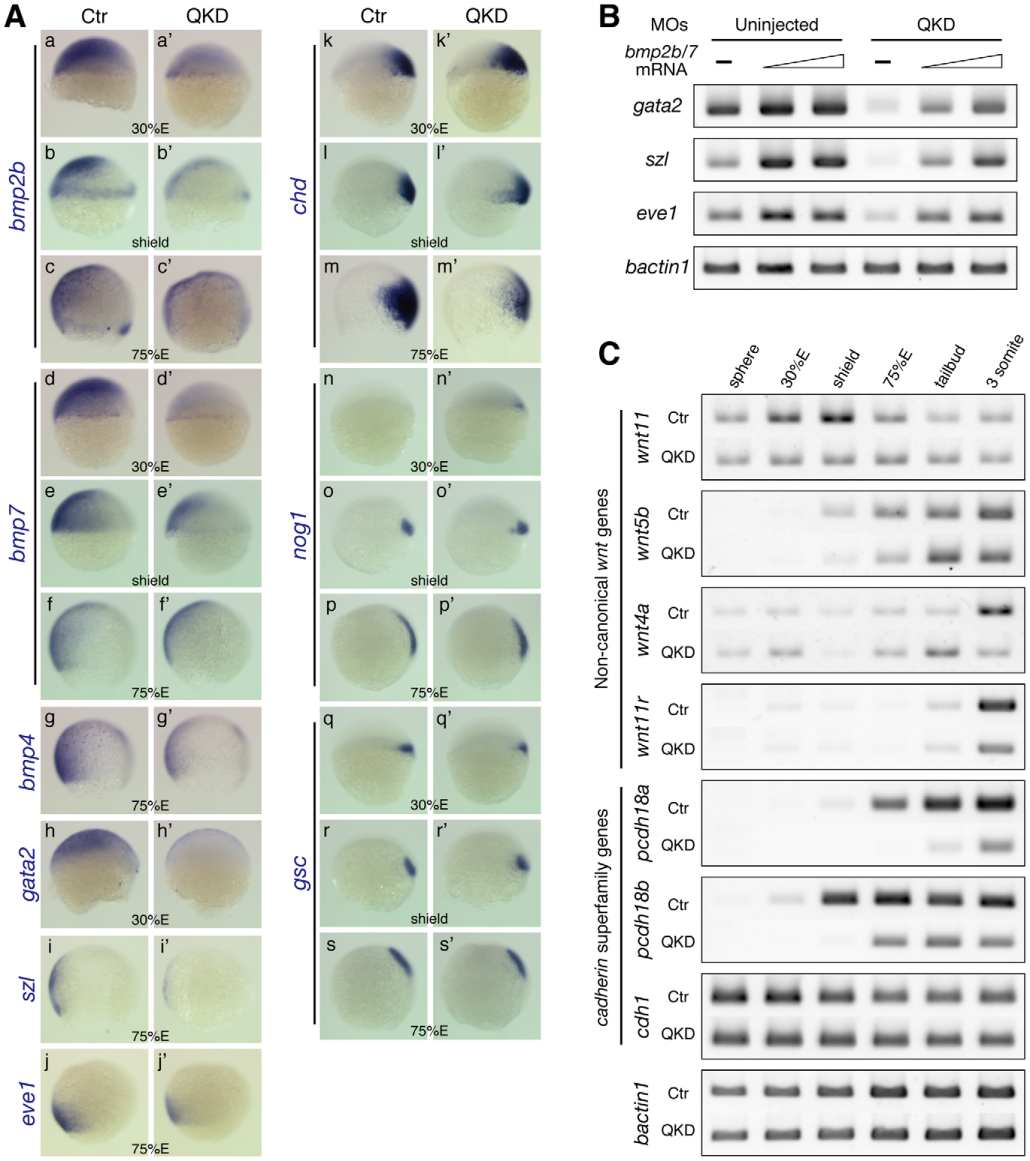
Defects in DV patterning and gastrulation movements in the QKD embryos. (A) DV patterning defects involving the reduced expression of *bmp* genes. Expression of *bmp2b/7/4* (a–g) and the Bmp downstream genes *gata2*, *szl* and *eve1* (h–j) is reduced in the QKD embryos. (k–s) Expression of the Bmp antagonist genes, *chd* and *nog1*, and the organizer gene *gsc*. All are lateral views with dorsal to the right. (B) The BMP downstream genes are restored by an exogenous supply of *bmp2b/7*. Embryos were injected together with the MOs for QKD and a mixture of *bmp2b/7* mRNAs (20 or 40 pg each) and subjected to RT-PCR analysis at the shield stage. *bactin1* was used as an RT-PCR control. (C) Decreased expression of genes regulating C&E movements in the QKD embryos. Temporal expression profiles of the indicated genes in the uninjected control and QKD embryos from the sphere to 3-somite stages were determined by RT-PCR. Expression of non-canonical *wnt* genes is reduced in the QKD embryos. Expression of *pcdh18a*/*18b* is also reduced in the QKD embryos, whereas *cdh1*, which is known to be involved in epiboly, is expressed at normal levels. *bactin1* was used as an RT-PCR control.

Early DV patterning of the embryo relies on a gradient of Bmp signaling, in which *bmp2b/7* play a major role in zebrafish [36], [37]. The phenotypic similarities between the QKD embryos and *bmp* pathway mutants described above prompted us to examine the genes in this pathway. Expression of *bmp2b/7* was found to be reduced in the QKD embryos from the beginning (Figure 4Aa–4Af). *bmp4* expression levels were more or less normal initially, but were downregulated at late epiboly stages in the QKD embryos (Figure 4Ag; data not shown). Consistently, expression of the *gata2*, *szl* and *eve1* genes, which are immediately downstream of Bmp signaling, was also reduced in the QKD embryos (Figure 4Ah–4Aj).

The dorsal identity of the zebrafish embryo requires activation of maternal β-catenin, which then activates expression of *gsc* and the Bmp antagonist genes *chd* and *nog1* at the dorsal side. In the QKD embryos, these genes were initiated normally, although the expression of *chd* was slightly ventrally expanded at 30%E (Figure 4Ak), which is likely secondary to the reduced expression of *bmp2b/7*. At later stages in the QKD embryos, however, *chd* expression was rather decreased (Figure 4Am), contrasting to the *bmp* pathway mutants [38].

To determine the relationship between the dorsalized phenotype of the QKD embryos and Bmp signaling, we injected a mixture of *bmp2b/7* mRNAs (20 or 40 pg each) together with the MOs for QKD. This *bmp2b/7* injection rescued the expression of the Bmp downstream genes *gata2*, *szl* and *eve1* (Figure 4B), indicating that signaling components acting downstream of Bmp2b/7 are not affected in the QKD embryos. Consistently, the mRNA levels of Bmp receptor genes (*acvrl1*, *bmpr1aa*, *bmpr1ab* and *bmpr1ba*) and *smad5* were found to be normal in the QKD embryos by microarray (GEO accession number GSE18830). These observations, together with the normal initiation of the dorsal pathway in the QKD embryos, indicate that the DV patterning defects of the QKD embryos primarily result from the reduction of *bmp2b/7* expression.

### B1 *sox* are required for proper gastrulation movements

Components of non-canonical Wnt signaling and cell adhesion molecules are major regulators of gastrulation movements, including epiboly and C&E movements [32], [39]. Since these movements are severely impaired in the QKD embryos, we investigated expression profiles of genes related to these processes. *wnt11* and *wnt5b* are major Wnt ligand genes involved in C&E movements [31], [32]. In the QKD embryos, upregulation of *wnt11* that normally occurs during early gastrulation was not observed and expression of *wnt5b* was slightly reduced at late epiboly stages (Figure 4C). We also observed decreased expression of *wnt11r* and *wnt4a*, which are important for convergence movements at later stages [40]. These data suggest that the reduction of non-canonical Wnt ligands contributes to the impairment of C&E movements.

Both classical cadherins and protocadherins are involved in gastrulation movements [39]. We found that expression of *pcdh18a*/*18b* was significantly reduced in the QKD embryos (Figure 4C). These genes are expressed in the epiblast at the shield stage and later in the neuroectoderm in an overlapping manner in normal embryos [41], [42]. To examine how reduced activity of Pcdh18a/18b affects embryogenesis, we knocked down these two genes. Although only mild gastrulation defects were observed when these genes were knocked down separately, simultaneous KD caused a delay in epiboly and also C&E defects (Figure S6; for *pcdh18a* single KD, see also [41]). Delayed epiboly has also been reported for the hypomorphic *cdh1* mutants [43], [44], but its expression was not altered in our QKD embryos (Figure 4C). These observations indicate that multiple mechanisms involved in gastrulation movements are simultaneously affected in the QKD embryos.

### Dependence of neuronal differentiation programs on B1 *sox* activity

**Figure 5:**
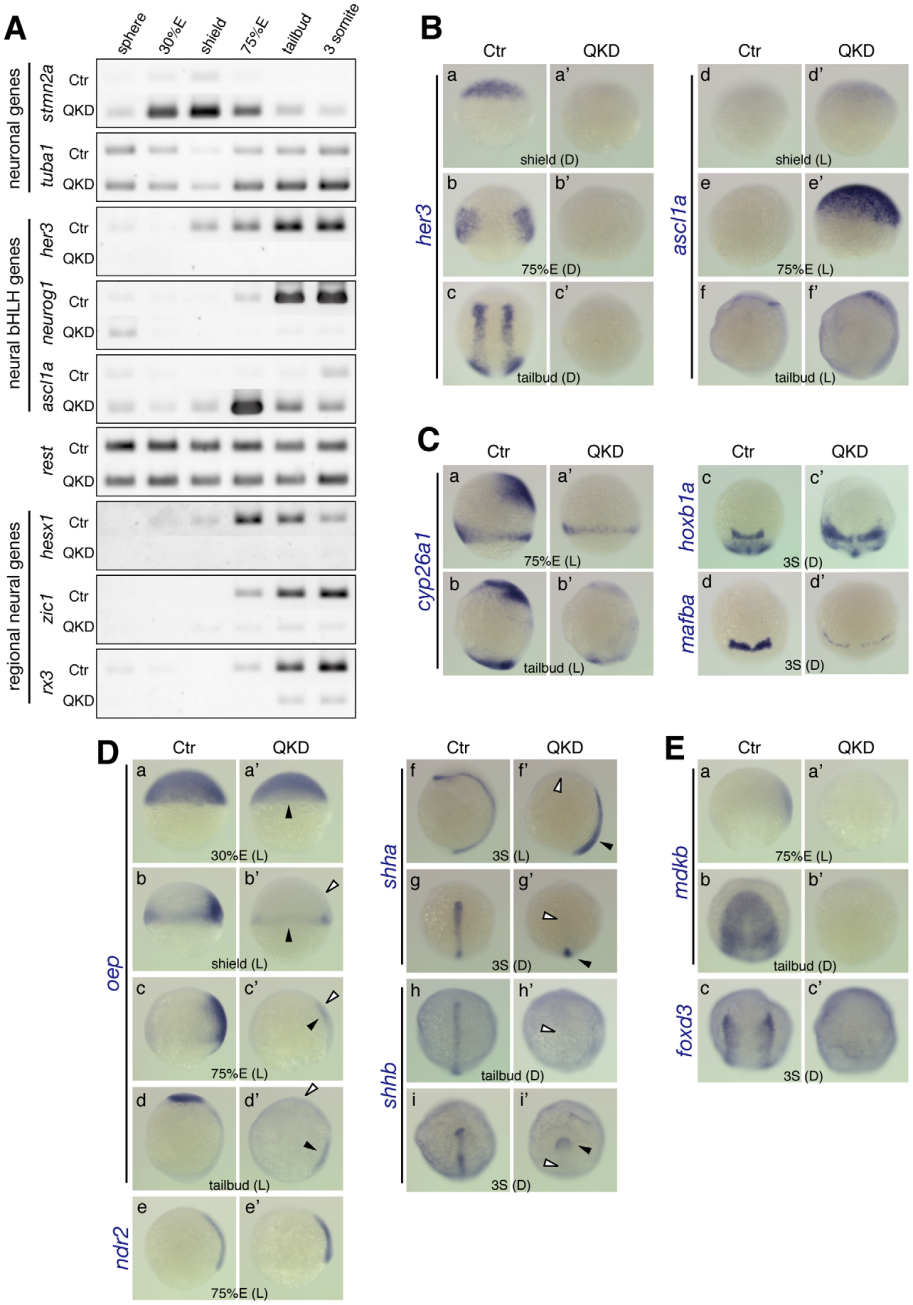
Impairment of neural development in the QKD embryos. (A) Altered expression of genes involved in neural differentiation. RT-PCR analysis of the indicated genes was performed as described in Figure 4C. (B) Effects of B1 *sox* QKD on neural bHLH gene expression. The *her3* expression is totally lost in the QKD embryos (a–c), whereas *ascl1a* is transiently upregulated at 75%E in a broad area of the neuroectoderm (d–f). (C) The loss of *cyp26a1* expression in the anterior neuroectoderm (a, b) and evidence of hindbrain patterning defects: expansion of *hoxb1a* expression (c) and a severe reduction of *mafba* expression (d). (D) The loss of expression of *oep* and *shha/b* in the neuroectoderm of the QKD embryos. (a–d) In the QKD embryos, the expression of *oep* is lost in the ectoderm at the shield stage (b) and the neuroectoderm at the 75%E and tailbud stages (c, d) (marked by open arrowheads in b’–d’), whereas its initial zygotic expression (a) and mesodermal expression (b–d) are maintained (closed arrowheads in a’–d’). (e) *ndr2* is also expressed in the QKD mesoderm at normal levels. (f–i) The expression of *shha/b* in the neuroectoderm is lost (marked by open arrowheads in f’–i’), whereas that in the mesoderm is retained (closed arrowheads in f’–i’). (E) The loss of *mdkb* expression in the neuroectoderm and severe reduction of *foxd3* expression in the neural crest cells of QKD embryos.

Several neuronal genes were found to be abnormally upregulated in the QKD embryos. *stmn2a* is strongly expressed in CNS neurons from mid-somitogenesis stages in wild-type embryos [45] and also weakly expressed throughout the embryo at epiboly stages (Figure 5A and Figure S7A). The latter early stage expression was found to be aberrantly upregulated in the QKD embryos (Figure 5A and Figure S7A). The neuronal *tuba1* gene was also upregulated from 75%E (Figure 5A). These observations suggest that a portion of the neuronal differentiation programs is precociously initiated in the QKD embryos.

Neural bHLH transcription factors are key players in the neuronal differentiation programs. *Hes/her* genes encode repressor-type bHLH proteins, are expressed in undifferentiated neural progenitor cells and maintain their cell state [46]. Among the zebrafish *her* genes, *her3*, an ortholog of mammalian *Hes3*, is initiated in the dorsal region of the epiblast at about 30%E, and its expression continues in bilateral inter-proneuronal domains [47]. This *her3* expression is totally lost in the QKD embryos (Figure 5A and 5Ba–5Bc).

Proneural genes encoding activator-type bHLH proteins and participating in neurogenesis are also affected in the QKD embryos. In normal zebrafish embryos, *neurog1* expression initially marks primary neurons at the end of gastrulation and then covers the proneuronal domains in a fashion complementary to *her3* expression. In the QKD embryos, *neurog1* expression is also lost (Figure 2Bf and Figure 5A). However, not all proneural genes behave in this manner as for example *ascl1a* is transiently upregulated at about 75%E in a broad area of the neuroectoderm in the QKD embryos (Figure 5A and 5B). Proneural genes are known to be repressed by Hes/Her [46], but exogenous injection of *her3* mRNA into QKD embryos did not repress aberrant *ascl1a* expression (data not shown), indicating that the loss of *her3* was not causal to this upregulation. The neuronal repressor REST has been implicated in suppression of *Ascl1* as well as *Stmn2* [48]. However, *rest* expression was unchanged in the QKD embryos (Figure 5A), although *Rest* is suggested to be downstream of SOX2 in ES cells [24], indicating that *rest* is not involved in the aberrant regulation of *ascl1a* and *stmn2a*.

Taken together, our results indicate that the proper operation of the neuronal differentiation programs, including regulatory networks involving the neural bHLH genes, is highly dependent on the activity of B1 *sox*.

### Involvement of B1 *sox* functions in neural patterning

Regional identities of the neural plate are specified through regulatory networks involving various signaling pathways and transcriptional regulators. Genes that are critical for these networks are severely affected in the QKD embryos. As described earlier, in the QKD embryos, expression of the transcription factor genes *hesx1*, *zic1*, and *rx3*, which are required for forebrain and eye development [49]–[51], is lost throughout early embryogenesis (Figure 3C and Figure 5A). The MHB itself was established, as judged from the expression of *pax2a* and *eng2a*, but the anterolaterally-shifted expression patterns of these genes suggest an improper formation of the axes of the anterior neural plate (Figure 2Bd and Figure 3Bf). The expression domain of *otx2* was expanded and encircled by those of *pax2a*, *eng2a* and *gbx1* (Figure 2Bd and Figure 3B), which is likely due to the dorsalized phenotype caused by the decreased expression of *bmp2b/7*, as the *bmp2b/7* mutant embryos show similar patterns of gene expression [33], [34]. In addition, the QKD embryos lacked the anterior neuroectoderm expression of *cyp26a1* (Figure 5C), which encodes an RA degrading enzyme and thereby plays a role in hindbrain patterning [52]. Hence, the reduction of *cyp26a1* expression partly accounts for the hindbrain defects in the QKD embryos, e.g., expansion of *hoxb1a* in r4 (Figure 5Cc) as observed in the *cyp26a1* mutant [53]. However, B1 *sox* also seem to be more directly involved in gene regulation in hindbrain development as evidenced by severe downregulation of *mafba* (r5/6) (Figure 5Cd) and the loss of *krox20* expression in r3/5 (Figure 3Ce).

Nodal and Sonic hedgehog signaling are crucial for the development of ventral brain structures [54]–[56]. In normal embryos, *oep*, an ortholog of mouse *Cripto*, is strongly expressed in the anterior neural plate and is essential as a coreceptor for receiving Nodal signals [54]. Interestingly, *oep* expression in the ectoderm at the shield stage and the neuroectoderm at later stages is selectively lost in the QKD embryos (Figure 5Db–5Dd), whereas its early zygotic and mesodermal expression was maintained (Figure 5Da–5Dd). Moreover, expression of *shha* and *shhb* in the ventral floor of the brain is also lost in the QKD embryos, leaving only *shhb* expression in the prechordal plate (Figure 5Df–5Di). These findings suggest that the loss of *oep* and *shha/b* expression leads to defective ventral brain development in the QKD embryos. It is known that *shha* expression in the neuroectoderm is regulated by Nodal signals from the mesoderm [57]. However, a more direct link between B1 SOX action and *shh* regulation is suggested, as the exogenous injection of *oep* mRNA into the QKD embryos did not restore the *shha* expression (data not shown), although Nodal-encoding *ndr2* is normally expressed (Figure 5De).

Defects in the anterior neural plate development in the QKD embryos also include the loss of *mdkb* expression (Figure 5E). Consistent with the proposed role of *mdkb* in the specification of neural crest cells [58], *foxd3* expression in the neural crest was reduced in the QKD embryos (Figure 5Ec).

### Regulatory actions of the B1 SOX proteins in early zebrafish embryos

**Figure 6:**
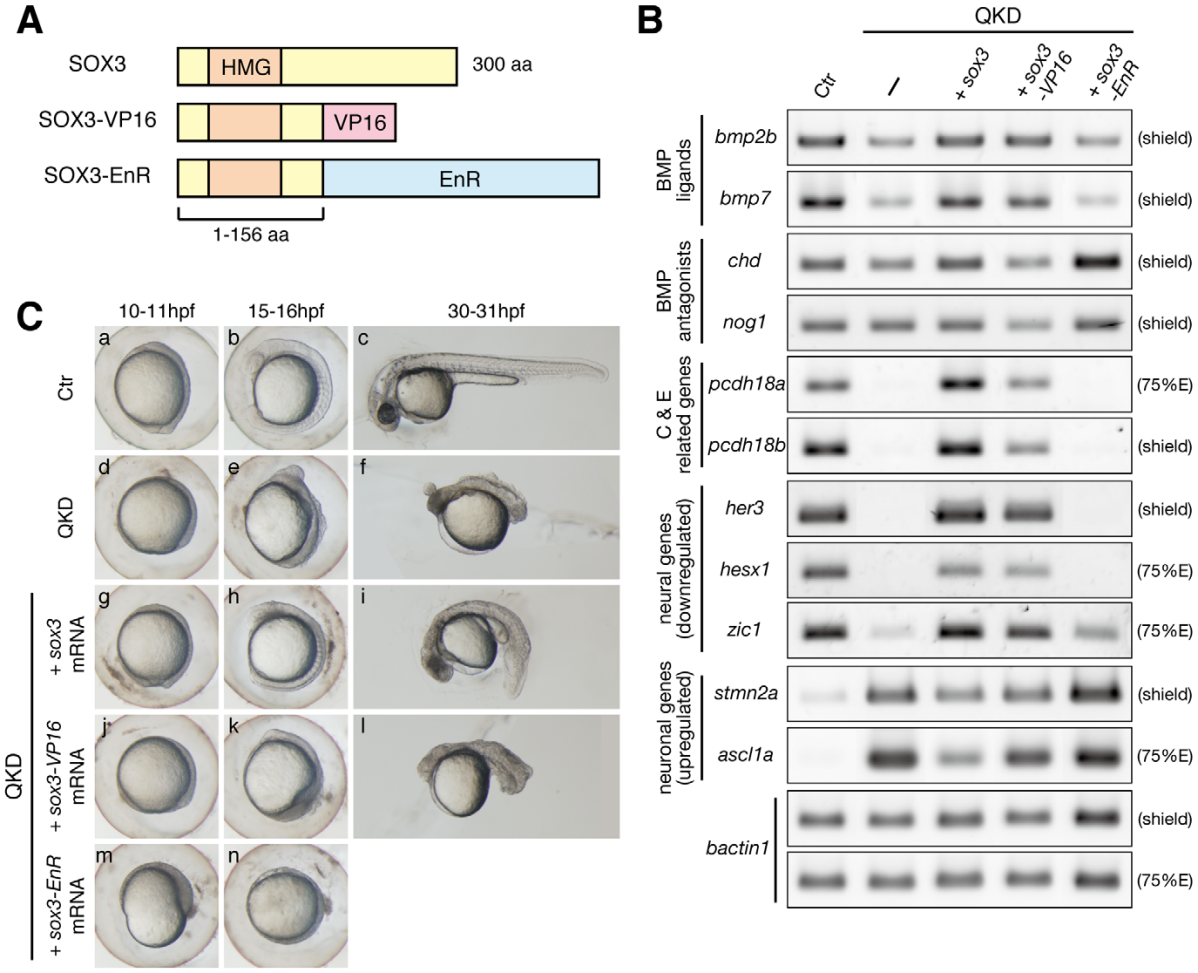
Regulatory actions of the B1 SOX proteins. (A) Schematic representation of the protein structures of SOX3, SOX3-VP16 and SOX3-EnR. Dominant activator and repressor forms of SOX3 were constructed by fusing the VP16 activation and Engrailed repression (EnR) domains, respectively, to truncated SOX3(1–156 aa). (B) Gene expression responses to SOX3, SOX3-VP16 or SOX3-EnR under QKD conditions. mRNAs of *sox3*, *sox3-VP16* and *sox3-EnR* (20 pg) were individually injected with the MOs for QKD and gene expression responses were examined by RT-PCR. The exogenous supply of either SOX3 or SOX3-VP16 but not by SOX3-EnR recovered expression of genes that were downregulated (*bmp2b/7*, *pcdh18a/18b*, *her3*, *hesx1* and *zic1*) in the QKD embryos and also suppressed expression of genes that were upregulated (*stmn2a* and *ascl1a*). *bactin1* was used as an RT-PCR control. (C) Partial rescue by SOX3-VP16 and strengthening by SOX3-EnR of the morphological phenotypes of the QKD embryos. Live embryo images at 10–11, 15–16, and 30–31 hpf were observed. SOX3-VP16-injected embryos showed a rather ventralized phenotype. Embryos coinjected with SOX3-EnR died during late segmentation stages. All are lateral views.

As the B1 SOX proteins primarily function as transcriptional activators [11], [13], [15], [23], [25], the expression of direct target genes is expected to be decreased in response to the QKD. However, the upregulation of several neuronal genes such as *stmn2a* raised the possibility that B1 SOX might also act as repressors. To test this, we utilized dominant activator and repressor forms of SOX3, SOX3-VP16 and SOX3-EnR (Figure 6A) and compared the effects of these variants under QKD conditions with those of SOX3. As anticipated, genes that were downregulated in the QKD embryos, namely *bmp2b/7*, *pcdh18a/18b*, *her3*, *hesx1* and *zic1*, were efficiently recovered by the exogenous supply of either SOX3 or SOX3-VP16 but not by SOX3-EnR (Figure 6B). These genes are thus likely activation targets of B1 SOX. In addition, the increased expression of *stmn2a* and *ascl1a* in the QKD embryos was suppressed in the same way. This suggests an indirect regulation of these genes by B1 SOX through the activation of repressors. However, SOX3-VP16 was less effective than SOX3 in the rescue of some genes such as *pcdh18a/18b* and *ascl1a*, suggesting that the activation process may require additional molecular interactions with the intact SOX3 protein.

It is noteworthy that the morphological rescue of the QKD embryos by SOX3-VP16 was much less complete when compared to that observed for SOX3, and that the SOX3-VP16-injected embryos showed a rather ventralized phenotype (Figure 6C). In line with this observation, *chd* and *nog1* were unexpectedly reduced in SOX3-VP16-injected embryos, but increased in SOX3-EnR-injected embryos, suggesting that the repressive action of B1 SOX may be required for the proper regulation of dorsally expressed BMP antagonist genes. These findings together indicate that the B1 SOX proteins primarily act as activators in early embryos, whereas a context-dependent repressive action of these factors is also suggested.

### Direct regulatory targets of B1 SOX in early zebrafish embryos

**Figure 7:**
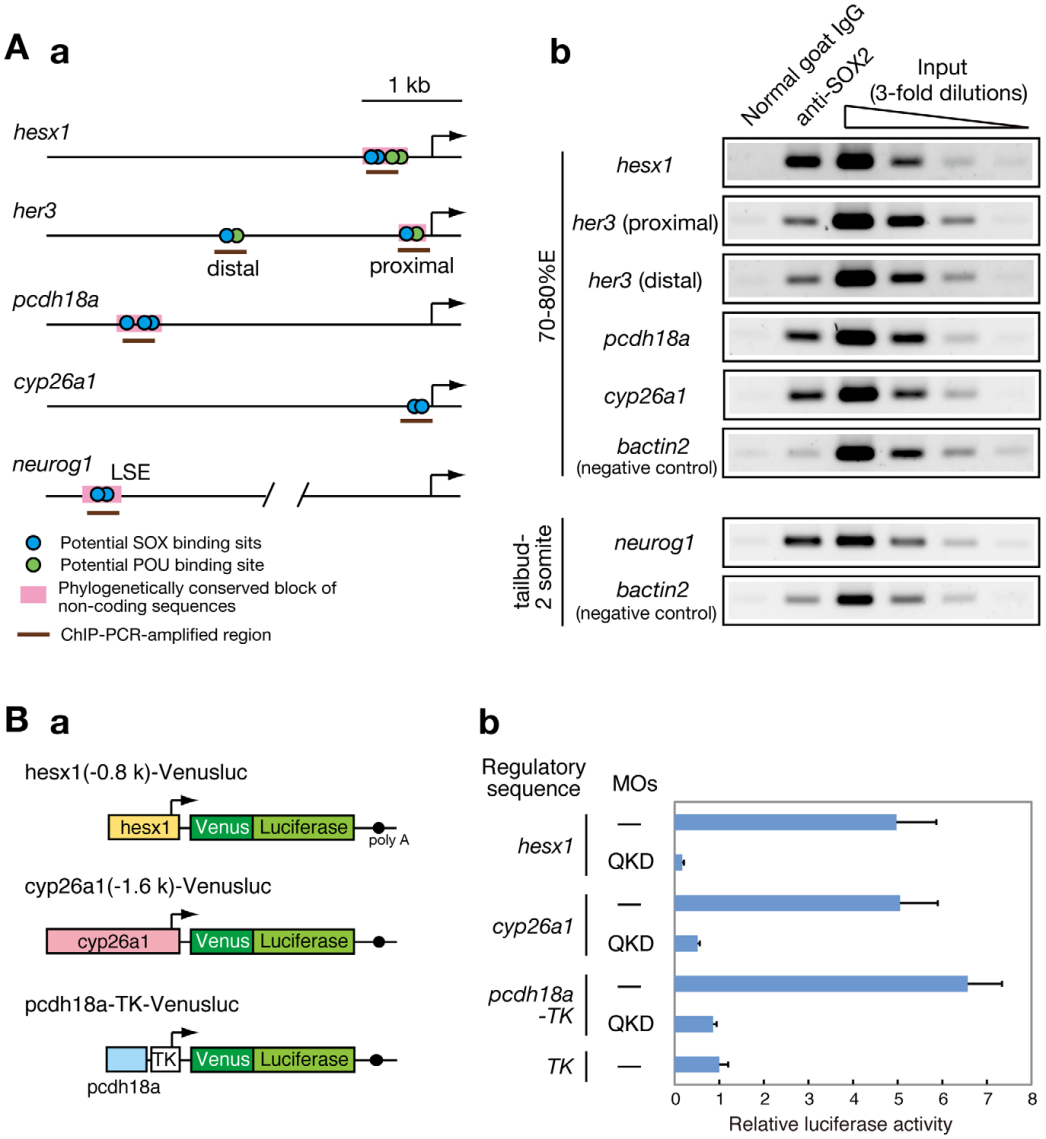
Direct regulatory targets of the B1 SOX proteins in the zebrafish embryo. (A) ChIP analysis showing direct association of B1 SOX with regulatory sequences of the downstream genes. (a) Potential binding sites for SOX and POU within the analyzed genomic regions are schematically shown. (b) ChIP-PCR analysis using anti-SOX2 antibody. ChIP experiments were performed using zebrafish embryos at the 70–80%E and tailbud to 2-somite stages. ChIP-PCR analysis using anti-SOX2 antibody that weakly cross-reacts with SOX3/19A/19B revealed specific binding of B1 SOX to the regulatory sequences of the *hesx1*, *her3*, *pcdh18a*, *cyp26a1* and *neurog1* genes in the zebrafish embryo. *bactin2* was used as a negative control. (B) B1 SOX-dependent activities of the regulatory sequences of *hesx1*, *cyp26a1* and *pcdh18a*. (a) The Venusluc fusion reporter (Venus plus firefly luciferase) constructs containing either of the promoters for *hesx1* or *cyp26a1* or the upstream conserved sequence of *pcdh18a* with the HSV TK promoter are schematically shown. (b) The Venusluc reporters were injected into embryos with or without the MOs for QKD together with the reference vector TK-*Renilla* luciferase. More than 20 injected embryos per sample were collected at the tailbud stage, and luciferase assays were performed. The normalized luciferase activity generated by TK-Venusluc was arbitrarily assigned a value of 1. Data are shown as the average values of four independent injection experiments with standard errors.

To further explore whether the B1 SOX proteins directly regulate the potential downstream genes described above, we searched for possible B1 SOX binding sites (containing the consensus sequence CATTGTT [21], [59] or closely related sequences) in the regulatory regions of these genes. We identified potential SOX-binding sites in the regulatory sequences of *her3* [47], *hesx1*, *cyp26a1* [60] and *neurog1* [61] and also in the conserved non-coding sequences upstream of *pcdh18a* (Figure 7Aa). To investigate the direct interaction of the B1 SOX proteins with these genomic sequences in vivo, ChIP experiments were performed using zebrafish embryos at the 70–80%E and tailbud to 2-somite stages. ChIP analysis using anti-SOX2 antibody that weakly cross-reacts with SOX3/19A/19B revealed specific binding of B1 SOX to these regulatory sequences in the zebrafish embryo (Figure 7Ab). Similar results were obtained with anti-SOX3 antibody (data not shown). It is thus likely that these genes are direct downstream targets of B1 SOX.

To further investigate whether the activities of these regulatory sequences are dependent on B1 SOX, we created luciferase reporter vectors containing the promoter sequences for *hesx1* and *cyp26a1* [60] (Figure 7Ba). The 0.8-kb promoter sequence of the zebrafish *hesx1* gene used here corresponds to the chicken *Hesx1* promoter that has been shown to have anterior CNS-specific regulatory activity in chicken and also zebrafish embryos [62]. The conserved non-coding sequence upstream of *pcdh18a* (412 bp) was also cloned into the TK-luciferase reporter vector (Figure 7Ba). When these reporter vectors were injected with or without the MOs for QKD into zebrafish embryos, the promoter activities of *hesx1* and *cyp26a1* were significantly downregulated upon B1 *sox* QKD (Figure 7Bb). The 412-bp *pcdh18a* sequence showed an enhancer activity in normal embryos, whereas this activity was also reduced in the QKD embryos (Figure 7Bb). These data confirm that B1 SOX proteins regulate the *hesx1*, *cyp26a1* and *pcdh18a* genes through these regulatory elements.

Interestingly, POU binding sites were found abutting the SOX sites of *her3* and *hesx1*. These genes were found to be commonly downregulated in the QKD embryos (Figure 3C and Figure 5A and 5B) and also MZ*spg* mutants (Table S7; see also [29]). In the QKD embryos, *pou5f1* is expressed at normal levels (Figure S7B), indicating that Pou5f1 alone is insufficient to induce *her3* or *hesx1*. These data together suggest that B1 SOX and Pou5f1 proteins synergistically cooperate to activate *her3* and *hesx1*.

## Discussion

**Figure 8:**
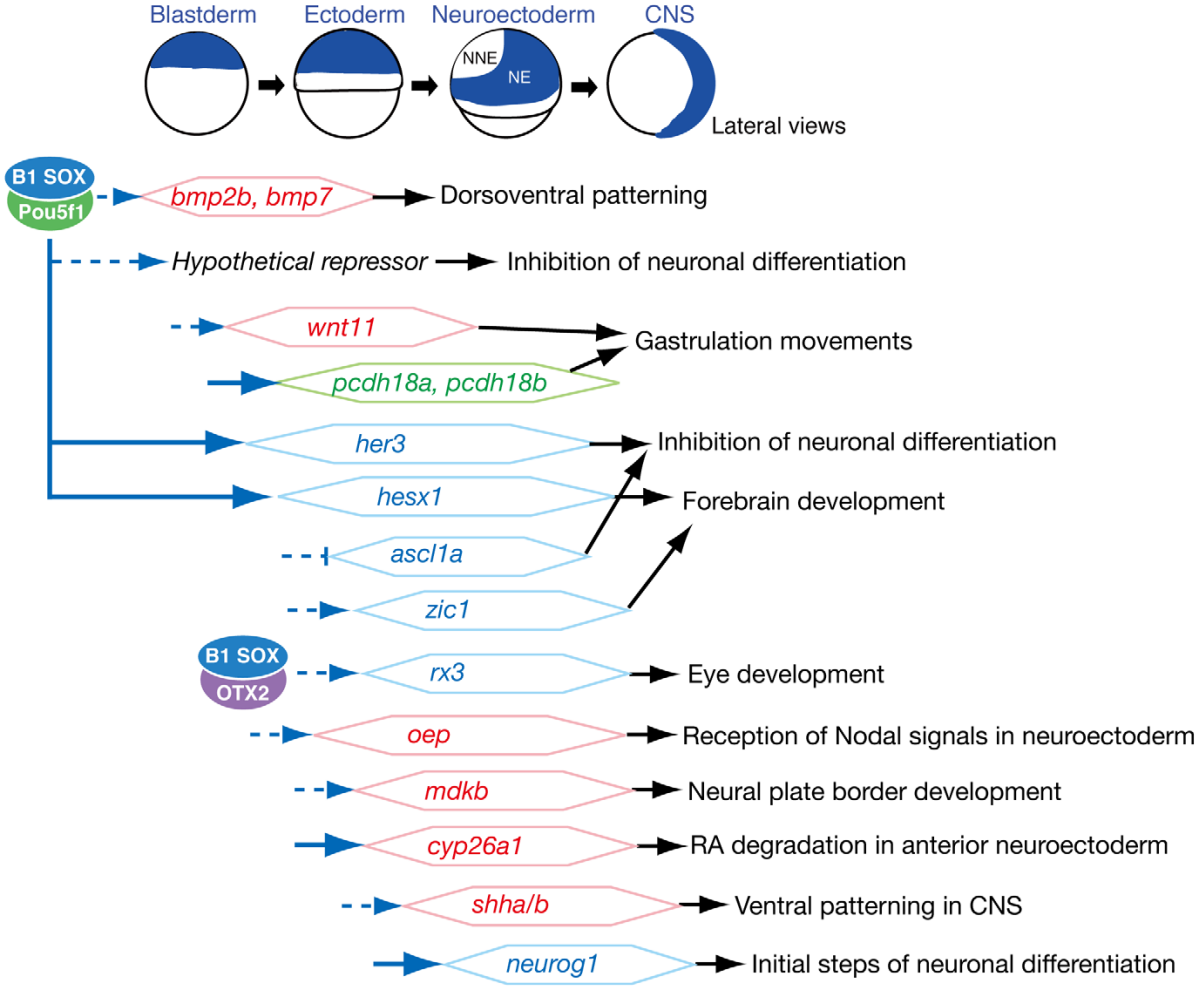
Summary of embryonic stage-dependent target gene regulation by B1 SOX. The B1 *sox* expression domains are schematically illustrated at the top. The B1 SOX-downstream genes that were identified in this study are shown with possible time windows for regulation. The major downstream genes of B1 SOX were found to be developmental transcription factor genes (indicated in blue), signaling pathway genes (red) and cell adhesion molecule genes (green). Our data indicate that, in these regulations, B1 SOX primarily act as activators and appear to indirectly repress several target genes through the activation of hypothetical repressors. A direct regulation of the *pcdh18a*, *her3*, *hesx1*, *cyp26a1* and *neurog1* genes by B1 SOX is suggested by our ChIP analysis (indicated by blue arrows). The developmental effects of the regulation by B1 SOX are indicated on the right.

Previous studies have suggested that the group B1 *sox* genes are critical for early processes in embryogenesis, particularly during early neural development [1]–[6]. Possibly as a consequence of functional redundancy, however, loss-of-function analyses have not been sufficiently informative to date. In our present study, we successfully depleted the B1 *sox* activity from early zebrafish embryos by a quadruple knockdown of *sox2/3/19a/19b*, and present clear evidence that the B1 *sox* genes are highly redundant and their encoding proteins are functionally interchangeable in early zebrafish embryogenesis. More importantly, we demonstrate that the B1 *sox* genes are indeed essential for several key processes during early embryogenesis, namely embryonic patterning, gastrulation movements and neural development. The major downstream genes of B1 SOX that function in these processes were found to be developmental transcription factor genes, signaling pathway genes and cell adhesion molecule genes (Figure 8). These data indicate that B1 SOX proteins play a central role in coordinating cell fate specification with embryo patterning and morphogenetic processes by controlling a wide variety of developmental regulators in a process-dependent manner. Among the broad functions of B1 SOX, the transcriptional partnership with Pou5f1 is critical for early embryogenesis from the blastoderm to early neural stages as detailed further below.

### The partnership of B1 SOX and Pou5f1, a homolog of mammalian Oct3/4, in early embryogenesis

We found similarities in the gene expression profiles between the B1 *sox* QKD embryos and the MZ*spg* embryos in a wider range of developmental stages from the blastoderm to early neural stages (Figure 8, Table S7 and [29]). This strongly suggests that their cooperation is required not only for the blastoderm stage, which may be similar to the ES cell state, but also for the early neural stage. In contrast to mouse knockouts of *Sox2* or *Oct3/4*, where impairment of the ICM/epiblast lineage development causes early embryonic lethality [1], [63], zebrafish embryos of the B1 *sox* QKD and MZ*spg* mutants are viable although with severe developmental defects. This enabled us to analyze the functions of B1 *sox* in later developmental stages as well as the blastoderm stage.

A group of key genes downstream of B1 SOX and Pou5f1 in the blastoderm was found to be *bmp2b/7*, as the expression of *bmp2b/7* is also severely reduced in MZ*spg* embryos [28]. In addition, an overlap of the expression domains of B1 *sox*, *pou5f1* and *bmp2b/7* in the blastoderm strongly suggests a direct regulation of *bmp2b/7* by B1 SOX and Pou5f1. The dorsalized phenotype of the B1 *sox* QKD and MZ*spg* embryos can to a large extent be ascribed to the *bmp2b/7* defects, as *bmp2b/7* mRNA injection into the QKD embryos rescues the expression of Bmp downstream genes (Figure 4B) and *bmp2b* mRNA injection can also rescue the MZ*spg* embryos [28]. This co-regulation thus appears to be critical for the establishment of the early DV axis, but likely operates only during the initial activation of *bmp* genes, since during gastrulation the expression domains of B1 *sox* and *bmp2b/7* segregate and eventually become complementary to each other.

*her3* and *hesx1*, which both encode transcriptional repressors, were identified as direct targets of B1 SOX in the early phase of neural development (Figure 5, Figure 6, and Figure 7). The expression of these genes is also lost in the MZ*spg* embryos (Table S7 and [29]). In addition, our ChIP analysis indicated that the proximal and distal SOX-POU elements of the *her3* promoter and the *hesx1* promoter carrying multiple SOX and POU sites are bound by B1 SOX in vivo (Figure 7A). We further verified, using a luciferase reporter assay, that the activity of *hesx1* promoter is dependent upon B1 SOX. These observations together indicate that *her3* and *hesx1* are regulated under the cooperative action of B1 SOX and Pou5f1. In addition, the B1 SOX and Pou5f1 complex appears to be required for expression of hypothetical repressors that inhibit neuronal differentiation, since the expression of *stmn2a* is aberrantly upregulated in the B1 *sox* QKD embryos and also in MZ*spg* embryos (Figure 5A, Figure S7A, and Table S7). These data indicate that in the early phases of neural development B1 SOX proteins cooperate with Pou5f1 and activate the transcriptional repressor genes that inhibit the further differentiation of neural progenitor cells.

### The role of the B1 *sox* genes in coordinating the developmental processes in the gastrulating embryo

In the gastrulating embryo, cell fate specification must be coupled with embryo patterning and gastrulation movements. We found that B1 SOX proteins are involved in all these processes by controlling their respective regulators in the gastrulating embryo.

In the early phase of neural fate specification, the B1 SOX proteins are required for the activation of *her3* and the repression of *ascl1a*, which by themselves are inhibitory to neuronal differentiation (Figure 8). On the other hand, B1 SOX also appear to play a role in an initial phase of neuronal differentiation by directly activating the proneural *neurog1* gene. *neurog1* is severely downregulated in the QKD embryos (Figure 2 and Figure 5), and in vivo binding of B1 SOX to its regulatory sequence LSE is indicated by our ChIP analysis. In addition, although B1 SOX proteins are known to counteract neurogenesis, this inhibition occurs at late steps of neurogenesis without affecting *Neurog1/2* expression [13]. Taken together, these data indicate that B1 SOX are important for the successive generation of neural progenitor cells and immature neuronal cells.

Another important aspect of the functions of the B1 *sox* genes during neural lineage differentiation is that the initiation of the transcription factor genes *zic1* and *rx3* depends on their activity (Figure 3 and Figure 5). *rx3* may also be directly regulated by B1 SOX, since *Xenopus Rx1*, a functional homolog of zebrafish *rx3*, is under the direct regulation of SOX2 and Otx2 in the eye field [64]. Zic1 and Rx3 generally act as a transcriptional activator and are required for forebrain and eye lineage development [50], [51].

Critical functions of B1 *sox* in embryo patterning are underscored by our present findings that the neural expression of the signaling pathway genes *cyp26a1*, *oep*, *shha/b* and *mdkb* is dependent upon B1 *sox* activity (Figure 5). Signaling pathways involving these genes play a key role in cell fate decisions as well as diverse patterning processes in the developing CNS [52]–[56], [58]. Our ChIP and promoter analyses suggest a direct regulation of *cyp26a1* by SOXB1 (Figure 7). Furthermore, the expression of *oep*, *mdkb* and *shha/b* extensively overlaps with that of B1 *sox* in the neuroectoderm, also implying direct regulation by B1 SOX. Interestingly, the expression of *Shh* during mouse hippocampal development has recently been shown to be directly regulated by SOX2 [65].

A remarkable defect of the QKD embryos was also found to occur in gastrulation movements. Delayed epiboly and impaired C&E movements are also shared phenotypes with the MZ*spg* embryos [26]–[28], suggesting that these processes may also be co-regulated by B1 SOX and Pou5f1. We speculate that a severe reduction of *pcdh18a/18b* in combination with a reduced expression of non-canonical *wnt* genes is largely responsible for the defects in epiboly and C&E movements of the QKD embryos (Figure 4). We have further shown in our present analyses that the conserved sequence block upstream of *pcdh18a* acts as a B1 SOX-dependent enhancer. The in vivo binding of B1 SOX to this enhancer indicated by our ChIP analysis further supports a direct regulation of *pcdh18a* by B1 SOX. The knockdown phenotypes of *pcdh18a/18b* were consistent with their important functions in gastrulation movements (Figure S6 and [41]), but their molecular role in this process is still unclear. Recent studies, however, have reinforced the critical role of cell adhesion molecules in gastrulation movements [39]. Pcdh8 (Papc), structurally similar to Pcdh18, controls C&E movements of the paraxial mesoderm in cooperation with the non-canonical Wnt pathway [66], suggesting analogous roles of Pcdh18a/18b in the ectoderm.

The findings of our current study thus demonstrate that the B1 SOX proteins regulate genes that are critical for a variety of processes in early embryonic development. This suggests that these factors serve as central coordinators of gene regulatory networks in the early developing embryo by coupling cell fate specification with patterning and morphogenetic processes. In transcriptional regulation, B1 SOX proteins likely perform this coordination by partnering with a variety of factors including Pou5f1 [21]. Our microarray analysis also suggests that B1 SOX regulate additional genes and pathways that we did not investigate herein. Future studies of these genes will therefore more fully delineate their multiple functions in coordinating early embryogenesis.

## Materials and Methods

### MO-mediated knockdowns

MOs were obtained from Gene Tools LLC (OR, USA) and are listed in Table S4. Zebrafish embryos were obtained by natural matings of wild-type TL fish and reared at 28.5°C in 0.03% Red Sea salt solution. Approximately 1 nl of solution containing various combinations of MOs, as indicated in the figures, was injected into 1-cell stage embryos. Unless otherwise noted, a 1∶1 mixture of two MOs (0.9 ng/nl each) was used to knockdown individual B1 *sox* genes. To knockdown multiple B1 *sox* genes, the MOs were each mixed at a concentration of 0.9 ng/nl and injected into 1-cell stage embryos.

To estimate the knockdown efficiency of B1 *sox* MOs, fusion mRNAs of *sox3-luc*, *sox19a-luc* and *sox19b-luc* were prepared by transcription of template vectors, in which the 5′-UTR sequence and a short stretch of the amino-terminal-coding sequence of the respective genes (−131 to +32 of *sox3*; −151 to +32 of *sox19a*; −151 to +32 of *sox19b*) were inserted upstream of the luciferase sequence as previously described [20]. mRNA were microinjected with the relevant MOs into embryos and luciferase activities expressed in the embryos at 10–11 hpf were measured as described [20].

### Gene expression analysis

Whole-mount in situ hybridizations of zebrafish embryos were performed as described previously [11]. The genes analyzed are listed in Table S1.

Total RNAs for RT-PCR and microarray analyses were prepared from 40–100 uninjected control embryos and an equivalent number of embryos injected with MOs and/or mRNAs using RiboPure kit (Ambion, TX, USA). 200 ng of total RNA of each sample was reverse-transcribed using oligo-dT primer and Superscript III RT-PCR system (Invitrogen, CA, USA) and a 1/80 fraction of the cDNA was used for PCR templates. PCR was performed using ExTaq polymerase (Takara, Japan) in 25 µl ExTaq buffer containing 5% dimethyl sulfoxide, 0.17 mM cresol red, 10% sucrose and primers listed in Table S5. The PCR temperature profile consisted of 5 min denaturation, 23–30 cycles of a 30 sec denaturation at 94°C, 30 sec annealing at 57–61°C and 15 sec primer extension at 72°C and lastly a 10 min extension at 72°C (see Table S5 for cycle numbers and annealing temperatures). PCR products were separated in a 2% agarose gel (1.5% Methaphor agarose/0.5% agarose) and stained with SYBR green I.

For microarray analysis, cRNA probes were prepared using 4 µg total RNA with a one-cycle cDNA synthesis kit (Affymetrix, CA, USA). Affymetrix Zebrafish Genome arrays were hybridized with 10 µg cRNA probes, and posthybridization staining and washing were performed according to the manufacturer's instructions. RNAs from two independent samples were analyzed for each embryonic stage and the data were processed using the RMA program. Fold changes of the averaged hybridization signals between control and QKD embryo samples were then determined (Figure S5, Table S2 and Table S3). The microarray data have been deposited in the Gene Expression Omnibus (GEO, http://www.ncbi.nlm.nih.gov/geo) at the National Center for Biotechnology Information with the accession number GSE18830.

### Western blotting

Western blotting with an anti-SOX2 antibody was carried out as described previously [20]. For the detection of SOX3, SOX19A and SOX19B, an anti-SOX3 C-terminal peptide antibody [15] was used. As a loading control, the blotted PVDF membranes were stained with Coomassie Brilliant Blue R-250.

### Synthetic mRNAs for embryo injection

The coding sequences of the B1 *sox* genes and their derivatives were cloned into the pCBA3 vector [20]. The coding sequence of *bmp7* was amplified by RT-PCR and cloned into pCBA3. The cDNA clone cb670 (Zebrafish International Resource Center [ZIRC], OR, UAS) was used as a template for *bmp2b* mRNA. mRNAs were transcribed in vitro from linearized vectors using the mMessage mMachine SP6 kit (B1 *sox* and *bmp7*) or mMessage mMachine T7 Ultra kit (*bmp2b*) (Ambion). For the rescue experiments, each mRNA was mixed with the MOs for QKD and injected into 1-cell stage embryos.

### Chromatin immunoprecipitation

ChIP was carried out as described previously [67] with minor modifications. Briefly, zebrafish embryos at the 70–80%E and tailbud to 2-somite stages were enzymatically dechorionated with Pronase and then fixed in 1% formaldehyde in embryo medium for 15 min at room temperature. For each immunoprecipitation experiment, approximately 200 fixed embryos were homogenized in cell lysis buffer and incubated for 15 min on ice. Nuclei were collected by centrifugation, resuspended in 200 µl of nuclei lysis buffer, incubated for 10 min on ice and then sonicated using Bioruptor (Cosmo Bio, Japan) to yield DNA fragments with an average size of 400–500 bases. The supernatant of the sonicated cells was diluted 10-fold with ChIP dilution buffer (50 mM Tris-HCl [pH 8.0], 167 mM NaCl, 1.1% Triton X-100, 0.11% sodium deoxycholate). 950 µl of the diluted lysate was then incubated overnight at 4°C with Protein G Dynabeads (Invitrogen) that had been prebound to 2 µg of anti-SOX2 antibody (AF2018; R&D, MN, USA). The same volume of the lysate was precipitated with normal goat IgG as a negative control. 100 µl of the lysate was used as an input control. Beads were washed four times with RIPA buffer and once with TE buffer containing 50 mM NaCl. Bound complexes were eluted from the beads and cross-links were reversed in 200 µl of elution buffer for six hours at 65°C. Eluted DNA was then purified by treatment with RNase A, followed by proteinase K digestion, phenol∶chloroform∶isoamyl alcohol extraction and ethanol precipitation. Precipitated DNA was resuspended in 30 µl of TE buffer and 1 µl of the DNA suspension was used as a template for ChIP-PCR, which was performed using ExTaq polymerase (Takara) in 20 µl ExTaq buffer containing 0.17 mM cresol red, 10% sucrose and the primers listed in Table S6. PCR products were separated in a 2% agarose gel (1.5% Methaphor agarose/0.5% agarose) and stained with SYBR green I.

### Regulatory sequence analysis of the *hesx1*, *cyp26a1*, and *pcdh18a* genes

The fragments of the zebrafish *hesx1* promoter (Zv8_NA6682:4150–4984, 835 bp), *cyp26a1* promoter (Zv8_chr12:9333416–9335090, 1.7 kbp) and *pcdh18a* conserved upstream sequence (Zv7_chr1:9017351–9017762, 412 bp) were amplified by PCR from the zebrafish genome using following primers (linker sequences that incorporate restriction sites are indicated by lowercase): *hesx1* promoter, gggagatctCGTCAAACTCTCCAAACGTGGAT and ggggtcgacCTCAAGTCCTTTAATTTAACTCCAACTG; *cyp26a1* promoter, gggagatctAGTATTCCCCGTCCCATTGC and ggggtcgacGTTGAAGCGCGCAACTGATC; and *pcdh18a*, gggatgcatAAGGCCCGTCCCAACTGAGGG and gggagatctCTACGTCTCAATCTCCCTGACAGA. The luciferase reporter vectors were constructed using pTK200-Venusluc/ISceI, which was generated by inserting an I-SceI site downstream of the reporter poly(A) sequence of pTK200-Venusluc [35]. The fragments of the *hesx1* promoter and *cyp26a1* promoter were inserted into the upstream region of the Venusluc sequence by replacing the TK promoter sequence. The pcdh18a-TK-Venusluc vector was constructed by inserting the 412-bp *pcdh18a* sequence upstream of the TK promoter.

To perform the luciferase assay, 17.5 pg of the Venusluc vectors and 2.5 pg of the reference *Renilla* luciferase vector phRG-TK (Promega, WI, USA) were co-injected into 1-cell stage zebrafish embryos. Half of the embryos were then subsequently injected with the MOs for B1 *sox* QKD to assess their effects upon the luciferase expression. Injected embryos were collected at the tail bud stage and luciferase assays were performed as described previously [20].

### Transgenic zebrafish lines

The NES30-TK200-nlsVenus/ISceI transgene was constructed by inserting the octamerized NES30 sequence, which is a 30-bp *nestin* enhancer core sequence composed of SOX and POU binding sites [15], into pTK200-nlsVenus/ISceI. pTK200-nlsVenus/ISceI is identical to pTK200-Venusluc/ISceI except that nlsVenus [35] was used as the reporter. A transgenic line was produced by the I-SceI meganuclease method as previously described [35].

## Supporting Information

Figure S1 Expression of group B1 *sox* genes in zebrafish embryos. (A) Expression of the *sox2*, *sox3*, *sox19a* and *sox19b* genes in zebrafish embryos at the sphere and 30%E stages, visualized by whole-mount in situ hybridization. (B) Expression profiles of the B1 *sox* genes in uninjected control (Ctr) and QKD embryos from the sphere to 3-somite stages determined by RT-PCR. The amplification cycles used are indicated on the right. *bactin1* was used as an RT-PCR control and is shown in Figure 4C.
(1.38 MB TIF)

Figure S2 Evaluation of the knockdown efficiency of MOs targeting *sox2*, *sox3*, *sox19a* and *sox19b*. (A) Quantitative evaluation of the knockdown efficiency of MOs targeting *sox3*, *sox19a* and *sox19b* using a luciferase assay. (a) Schematic representation of sox3-luc, sox19a-luc and sox19b-luc fusion RNA constructs. (b–d) Inhibition levels caused by the injection of sox3-MOs (b), sox19a-MOs (c) and sox19b-MOs (d) were quantitatively measured using the luciferase assay-based system. Luciferase activity was measured using more than 20 injected embryos of the tailbud to early somite stages per sample. The luciferase activity generated by each sox-luc fusion in the absence of MOs was arbitrarily assigned a value of 100. Data are shown as the average values of three independent injection experiments with standard errors. (B) Inhibition of endogenous B1 SOX expression analyzed by western blotting. Lysates for SDS-PAGE were prepared using tailbud to early somite stage embryos that had been injected with the indicated MOs. The knockdown conditions were the same as those described in Figure 1. A seven-embryo equivalent amount of the lysate was used per lane. Western blotting was performed using an anti-SOX2 antibody that weekly cross-reacts with SOX3/19A/19B (a) and an anti-SOX3 antibody that preferentially detects SOX3/19A/19B (b). Note that the SOX19B expression levels are low at these stages.
(0.96 MB TIF)

Figure S3 B1 *sox* activity is efficiently eliminated from the zebrafish embryo by B1 *sox* QKD. (A) Schematic representation of the NES30-TK200-nlsVenus/ISceI transgene construct used. NES30 is the 30-bp *nestin* enhancer core sequence, which is composed of SOX and POU binding sites. (B) NES30-driven nlsVenus expression (controls in upper panels) was abolished by injection of the MOs for QKD (lower panels), confirming effective depletion of B1 SOX activity from the embryo.
(0.67 MB TIF)

Figure S4 Effects of the B1 *sox* QKD revealed using transgenic lines with the GAL4-medeated reporter expression in the CNS and axial mesoderm. (A) Effects of QKD on CNS development were examined using the GAL4 enhancer trap line, which reports the activity of *nr2f2* (*couptf*β) neural enhancers. Double transgenic embryos harboring GAL4VPmad2 (#5m-3) and UAS:DsRedExDR normally show reporter expression in the CNS, which mimics neural *nr2f2* expression. Injection of the MOs for QKD abolished this reporter expression, indicating impairment of CNS development. (B) Effects of QKD on axial mesoderm development, examined using the GAL4VP16(hg/nc) line. Double transgenic embryos harboring the GAL4VP16(hg/nc) transgene and UAS:DsRedEx show reporter expression in the hatching gland (hg) and notochord (nc). Strong expression of UAS:DsRedEx was observed after 1 dpf in this line. Injection of the MOs for QKD did not reduce reporter expression, indicating normal axial mesoderm differentiation in the morphants. However, hatching gland cells remained as a single ball-like structure in the heads of the severe morphants (arrowhead).
(1.12 MB TIF)

Figure S5 Gene expression profiles of the QKD embryos analyzed by microarray. Venn diagrams of genes that were found to be downregulated (A) and upregulated (B) in the B1 *sox* QKD embryos. Microarray analysis was carried out to compare gene expression profiles at the 30%E, 75%E, and tailbud stages between wild-type embryos and the QKD embryos. The numbers of Affymetrix zebrafish microarray probes that showed more than a twofold decrease (Table S2) or increase (Table S3) in the QKD embryos are shown. Annotated genes that were altered in all three stages are listed on the bottom in order of fold change at 75%E.
(0.42 MB TIF)

Figure S6 Phenotypes of *pcdh18a* and *pcdh18b* knockdowns. Bright-field images of live embryos at 10–11.5 hpf and 31–31.5 hpf are shown. (A) Uninjected control (Ctr) embryos. (B–D) Single and double knockdowns of *pcdh18a/18b*. Moderate amounts of MO (3.6 ng) were used for single KD (B,C) and a 1∶1 mixture of two MOs (3.6 ng each) was injected for double KD (D). Double knockdown embryos showed a shorter anteroposterior axis than the single knockdown embryos, indicative of more severe defects in C&E movements and suggesting a degree of functional redundancy between *pcdh18a* and *pcdh18b*.
(1.83 MB TIF)

Figure S7 Expression of *stmn2a* and *pou5f1* in the QKD embryos. (A) Expression of the *stmn2a* gene is upregulated in the QKD embryos at the shield stage. (B) Expression levels of *pou5f1* were not affected in the B1 *sox* QKD embryos (our microarray data), whereas its expression domain was ventrally expanded at 75%E.
(1.11 MB TIF)

Table S1 Summary of gene expression analysis using in situ hybridization and/or RT-PCR.
(0.39 MB DOC)

Table S2 Downregulated genes at 30% epiboly, 75% epiboly, and tailbud stages.
(0.13 MB XLS)

Table S3 Upregulated genes at 30% epiboly, 75% epiboly, and tailbud stages.
(0.12 MB XLS)

Table S4 Morpholino antisense oligonucleotides.
(0.11 MB DOC)

Table S5 Primers and conditions for RT-PCR.
(0.08 MB DOC)

Table S6 Primers and conditions for ChIP-PCR.
(0.04 MB DOC)

Table S7 Comparison of B1 SOX and Pou5f1 regulated genes.
(0.07 MB XLS)

## References

[1] Avilion AA, Nicolis SK, Pevny LH, Perez L, Vivian N (2003). Multipotent cell lineages in early mouse development depend on SOX2 function.. Genes Dev.

[2] Kishi M, Mizuseki K, Sasai N, Yamazaki H, Shiota K (2000). Requirement of Sox2-mediated signaling for differentiation of early *Xenopus* neuroectoderm.. Development.

[3] Mizuseki K, Kishi M, Matsui M, Nakanishi S, Sasai Y (1998). *Xenopus* Zic-related-1 and Sox-2, two factors induced by chordin, have distinct activities in the initiation of neural induction.. Development.

[4] Papanayotou C, Mey A, Birot AM, Saka Y, Boast S (2008). A mechanism regulating the onset of *Sox2* expression in the embryonic neural plate.. PLoS Biol.

[5] Takemoto T, Uchikawa M, Kamachi Y, Kondoh H (2006). Convergence of Wnt and FGF signals in the genesis of posterior neural plate through activation of the *Sox2* enhancer N-1.. Development.

[6] Uchikawa M, Ishida Y, Takemoto T, Kamachi Y, Kondoh H (2003). Functional analysis of chicken *Sox2* enhancers highlights an array of diverse regulatory elements that are conserved in mammals.. Dev Cell.

[7] Zorn AM, Barish GD, Williams BO, Lavender P, Klymkowsky MW (1999). Regulation of Wnt signaling by Sox proteins: XSox17α/β and XSox3 physically interact with β-catenin.. Mol Cell.

[8] Gontan C, de Munck A, Vermeij M, Grosveld F, Tibboel D (2008). Sox2 is important for two crucial processes in lung development: branching morphogenesis and epithelial cell differentiation.. Dev Biol.

[9] Matsumata M, Uchikawa M, Kamachi Y, Kondoh H (2005). Multiple *N-cadherin* enhancers identified by systematic functional screening indicate its Group B1 SOX-dependent regulation in neural and placodal development.. Dev Biol.

[10] Que J, Okubo T, Goldenring JR, Nam KT, Kurotani R (2007). Multiple dose-dependent roles for Sox2 in the patterning and differentiation of anterior foregut endoderm.. Development.

[11] Okuda Y, Yoda H, Uchikawa M, Furutani-Seiki M, Takeda H (2006). Comparative genomic and expression analysis of group B1 *sox* genes in zebrafish indicates their diversification during vertebrate evolution.. Dev Dyn.

[12] Kamachi Y, Iwafuchi M, Okuda Y, Takemoto T, Uchikawa M (2009). Evolution of non-coding regulatory sequences involved in the developmental process: reflection of differential employment of paralogous genes as highlighted by *Sox2* and group B1 *Sox* genes.. Proc Jpn Acad Ser B Phys Biol Sci.

[13] Bylund M, Andersson E, Novitch BG, Muhr J (2003). Vertebrate neurogenesis is counteracted by Sox1-3 activity.. Nat Neurosci.

[14] Graham V, Khudyakov J, Ellis P, Pevny L (2003). SOX2 functions to maintain neural progenitor identity.. Neuron.

[15] Tanaka S, Kamachi Y, Tanouchi A, Hamada H, Jing N (2004). Interplay of SOX and POU factors in regulation of the *Nestin* gene in neural primordial cells.. Mol Cell Biol.

[16] Nishiguchi S, Wood H, Kondoh H, Lovell-Badge R, Episkopou V (1998). *Sox1* directly regulates the γ-crystallin genes and is essential for lens development in mice.. Genes Dev.

[17] Rizzoti K, Brunelli S, Carmignac D, Thomas PQ, Robinson IC (2004). SOX3 is required during the formation of the hypothalamo-pituitary axis.. Nat Genet.

[18] Weiss J, Meeks JJ, Hurley L, Raverot G, Frassetto A (2003). *Sox3* is required for gonadal function, but not sex determination, in males and females.. Mol Cell Biol.

[19] Dee CT, Hirst CS, Shih YH, Tripathi VB, Patient RK (2008). Sox3 regulates both neural fate and differentiation in the zebrafish ectoderm.. Dev Biol.

[20] Kamachi Y, Okuda Y, Kondoh H (2008). Quantitative assessment of the knockdown efficiency of morpholino antisense oligonucleotides in zebrafish embryos using a luciferase assay.. Genesis.

[21] Kondoh H, Kamachi Y (2010). SOX-partner code for cell specification: Regulatory target selection and underlying molecular mechanisms.. Int J Biochem Cell Biol.

[22] Masui S, Nakatake Y, Toyooka Y, Shimosato D, Yagi R (2007). Pluripotency governed by *Sox2* via regulation of *Oct3/4* expression in mouse embryonic stem cells.. Nat Cell Biol.

[23] Yuan H, Corbi N, Basilico C, Dailey L (1995). Developmental-specific activity of the FGF-4 enhancer requires the synergistic action of Sox2 and Oct-3.. Genes Dev.

[24] Boyer LA, Lee TI, Cole MF, Johnstone SE, Levine SS (2005). Core transcriptional regulatory circuitry in human embryonic stem cells.. Cell.

[25] Kamachi Y, Uchikawa M, Tanouchi A, Sekido R, Kondoh H (2001). Pax6 and SOX2 form a co-DNA-binding partner complex that regulates initiation of lens development.. Genes Dev.

[26] Lachnit M, Kur E, Driever W (2008). Alterations of the cytoskeleton in all three embryonic lineages contribute to the epiboly defect of Pou5f1/Oct4 deficient MZ*spg* zebrafish embryos.. Dev Biol.

[27] Lunde K, Belting HG, Driever W (2004). Zebrafish *pou5f1/pou2*, homolog of mammalian *Oct4*, functions in the endoderm specification cascade.. Curr Biol.

[28] Reim G, Brand M (2006). Maternal control of vertebrate dorsoventral axis formation and epiboly by the POU domain protein Spg/Pou2/Oct4.. Development.

[29] Onichtchouk D, Geier F, Polok B, Messerschmidt D, Mössner R (2010). Zebrafish Pou5f1-dependent transcriptional networks in temporal control of early development.. Mol Syst Bio.

[30] Robu ME, Larson JD, Nasevicius A, Beiraghi S, Brenner C (2007). p53 activation by knockdown technologies.. PLoS Genet.

[31] Heisenberg CP, Tada M, Rauch GJ, Saude L, Concha ML (2000). Silberblick/Wnt11 mediates convergent extension movements during zebrafish gastrulation.. Nature.

[32] Myers DC, Sepich DS, Solnica-Krezel L (2002). Convergence and extension in vertebrate gastrulae: cell movements according to or in search of identity?. Trends Genet.

[33] Nguyen VH, Schmid B, Trout J, Connors SA, Ekker M (1998). Ventral and lateral regions of the zebrafish gastrula, including the neural crest progenitors, are established by a *bmp2b/swirl* pathway of genes.. Dev Biol.

[34] Nguyen VH, Trout J, Connors SA, Andermann P, Weinberg E (2000). Dorsal and intermediate neuronal cell types of the spinal cord are established by a BMP signaling pathway.. Development.

[35] Ogura E, Okuda Y, Kondoh H, Kamachi Y (2009). Adaptation of GAL4 activators for GAL4 enhancer trapping in zebrafish.. Dev Dyn.

[36] Little SC, Mullins MC (2009). Bone morphogenetic protein heterodimers assemble heteromeric type I receptor complexes to pattern the dorsoventral axis.. Nat Cell Biol.

[37] Schmid B, Furthauer M, Connors SA, Trout J, Thisse B (2000). Equivalent genetic roles for *bmp7/snailhouse* and *bmp2b/swirl* in dorsoventral pattern formation.. Development.

[38] Miller-Bertoglio VE, Fisher S, Sanchez A, Mullins MC, Halpern ME (1997). Differential regulation of *chordin* expression domains in mutant zebrafish.. Dev Biol.

[39] Hammerschmidt M, Wedlich D (2008). Regulated adhesion as a driving force of gastrulation movements.. Development.

[40] Matsui T, Raya A, Kawakami Y, Callol-Massot C, Capdevila J (2005). Noncanonical Wnt signaling regulates midline convergence of organ primordia during zebrafish development.. Genes Dev.

[41] Aamar E, Dawid IB (2008). Protocadherin-18a has a role in cell adhesion, behavior and migration in zebrafish development.. Dev Biol.

[42] Kubota F, Murakami T, Tajika Y, Yorifuji H (2008). Expression of protocadherin 18 in the CNS and pharyngeal arches of zebrafish embryos.. Int J Dev Biol.

[43] Kane DA, McFarland KN, Warga RM (2005). Mutations in *half baked*/E-cadherin block cell behaviors that are necessary for teleost epiboly.. Development.

[44] Shimizu T, Yabe T, Muraoka O, Yonemura S, Aramaki S (2005). E-cadherin is required for gastrulation cell movements in zebrafish.. Mech Dev.

[45] Burzynski GM, Delalande JM, Shepherd I (2009). Characterization of spatial and temporal expression pattern of SCG10 during zebrafish development.. Gene Expr Patterns.

[46] Kageyama R, Ohtsuka T, Kobayashi T (2008). Roles of *Hes* genes in neural development.. Dev Growth Differ.

[47] Hans S, Scheer N, Riedl I, v Weizsacker E, Blader P (2004). *her3*, a zebrafish member of the hairy-E(spl) family, is repressed by Notch signalling.. Development.

[48] Ballas N, Grunseich C, Lu DD, Speh JC, Mandel G (2005). REST and its corepressors mediate plasticity of neuronal gene chromatin throughout neurogenesis.. Cell.

[49] Andoniadou CL, Signore M, Sajedi E, Gaston-Massuet C, Kelberman D (2007). Lack of the murine homeobox gene *Hesx1* leads to a posterior transformation of the anterior forebrain.. Development.

[50] Loosli F, Staub W, Finger-Baier KC, Ober EA, Verkade H (2003). Loss of eyes in zebrafish caused by mutation of *chokh/rx3*.. EMBO Rep.

[51] Maurus D, Harris WA (2009). Zic-associated holoprosencephaly: zebrafish Zic1 controls midline formation and forebrain patterning by regulating Nodal, Hedgehog, and retinoic acid signaling.. Genes Dev.

[52] White RJ, Schilling TF (2008). How degrading: Cyp26s in hindbrain development.. Dev Dyn.

[53] Hernandez RE, Putzke AP, Myers JP, Margaretha L, Moens CB (2007). Cyp26 enzymes generate the retinoic acid response pattern necessary for hindbrain development.. Development.

[54] Gritsman K, Zhang J, Cheng S, Heckscher E, Talbot WS (1999). The EGF-CFC protein one-eyed pinhead is essential for Nodal signaling.. Cell.

[55] Mathieu J, Barth A, Rosa FM, Wilson SW, Peyrieras N (2002). Distinct and cooperative roles for Nodal and Hedgehog signals during hypothalamic development.. Development.

[56] Rohr KB, Barth KA, Varga ZM, Wilson SW (2001). The Nodal pathway acts upstream of Hedgehog signaling to specify ventral telencephalic identity.. Neuron.

[57] Müller F, Albert S, Blader P, Fischer N, Hallonet M (2000). Direct action of the Nodal-related signal Cyclops in induction of *sonic hedgehog* in the ventral midline of the CNS.. Development.

[58] Liedtke D, Winkler C (2008). Midkine-b regulates cell specification at the neural plate border in zebrafish.. Dev Dyn.

[59] Chakravarthy H, Boer B, Desler M, Mallanna SK, McKeithan TW (2008). Identification of DPPA4 and other genes as putative Sox2:Oct-3/4 target genes using a combination of in silico analysis and transcription-based assays.. J Cell Physiol.

[60] Hu P, Tian M, Bao J, Xing G, Gu X (2008). Retinoid regulation of the zebrafish *cyp26a1* promoter.. Dev Dyn.

[61] Blader P, Plessy C, Strähle U (2003). Multiple regulatory elements with spatially and temporally distinct activities control *neurogenin1* expression in primary neurons of the zebrafish embryo.. Mech Dev.

[62] Spieler D, Baumer N, Stebler J, Koprunner M, Reichman-Fried M (2004). Involvement of Pax6 and Otx2 in the forebrain-specific regulation of the vertebrate homeobox gene *ANF/Hesx1*.. Dev Biol.

[63] Nichols J, Zevnik B, Anastassiadis K, Niwa H, Klewe-Nebenius D (1998). Formation of pluripotent stem cells in the mammalian embryo depends on the POU transcription factor Oct4.. Cell.

[64] Danno H, Michiue T, Hitachi K, Yukita A, Ishiura S (2008). Molecular links among the causative genes for ocular malformation: Otx2 and Sox2 coregulate *Rax* expression.. Proc Natl Acad Sci U S A.

[65] Favaro R, Valotta M, Ferri AL, Latorre E, Mariani J (2009). Hippocampal development and neural stem cell maintenance require *Sox2*-dependent regulation of *Shh*.. Nat Neurosci.

[66] Unterseher F, Hefele JA, Giehl K, De Robertis EM, Wedlich D (2004). Paraxial protocadherin coordinates cell polarity during convergent extension via Rho A and JNK.. EMBO J.

[67] Wardle FC, Odom DT, Bell GW, Yuan B, Danford TW (2006). Zebrafish promoter microarrays identify actively transcribed embryonic genes.. Genome Biol.

