# Supplementary figures and images for: B1 SOX Coordinate Cell Specification with Patterning and Morphogenesis in the Early Zebrafish Embryo
Source: PLoS Genet. 2010 May 6;6(5):e1000936. doi: 10.1371/journal.pgen.1000936 (PMC2865518; doi:10.1371/journal.pgen.1000936)

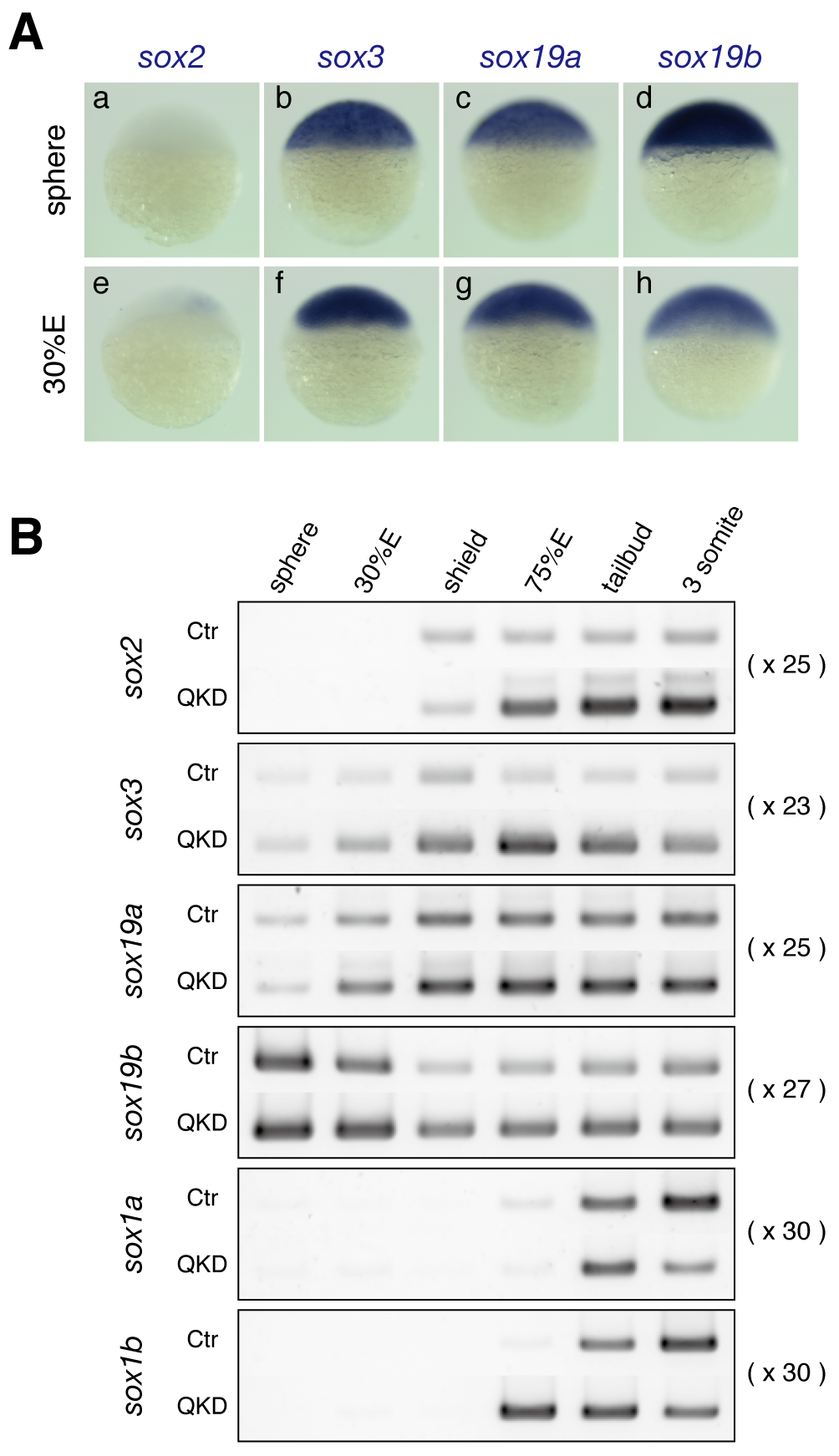

Supplement: Figure S1 — Expression of group B1 sox genes in zebrafish embryos. (A) Expression of the sox2, sox3, sox19a and sox19b genes in zebrafish embryos at the sphere and 30%E stages, visualized by whole-mount in situ hybridization. (B) Expression profiles of the B1 sox genes in uninjected control (Ctr) and QKD embryos from the sphere to 3-somite stages determined by RT-PCR. The amplification cycles used are indicated on the right. bactin1 was used as an RT-PCR control and is shown in Figure 4C. (1.38 MB TIF) [file pgen.1000936.s001.tif]

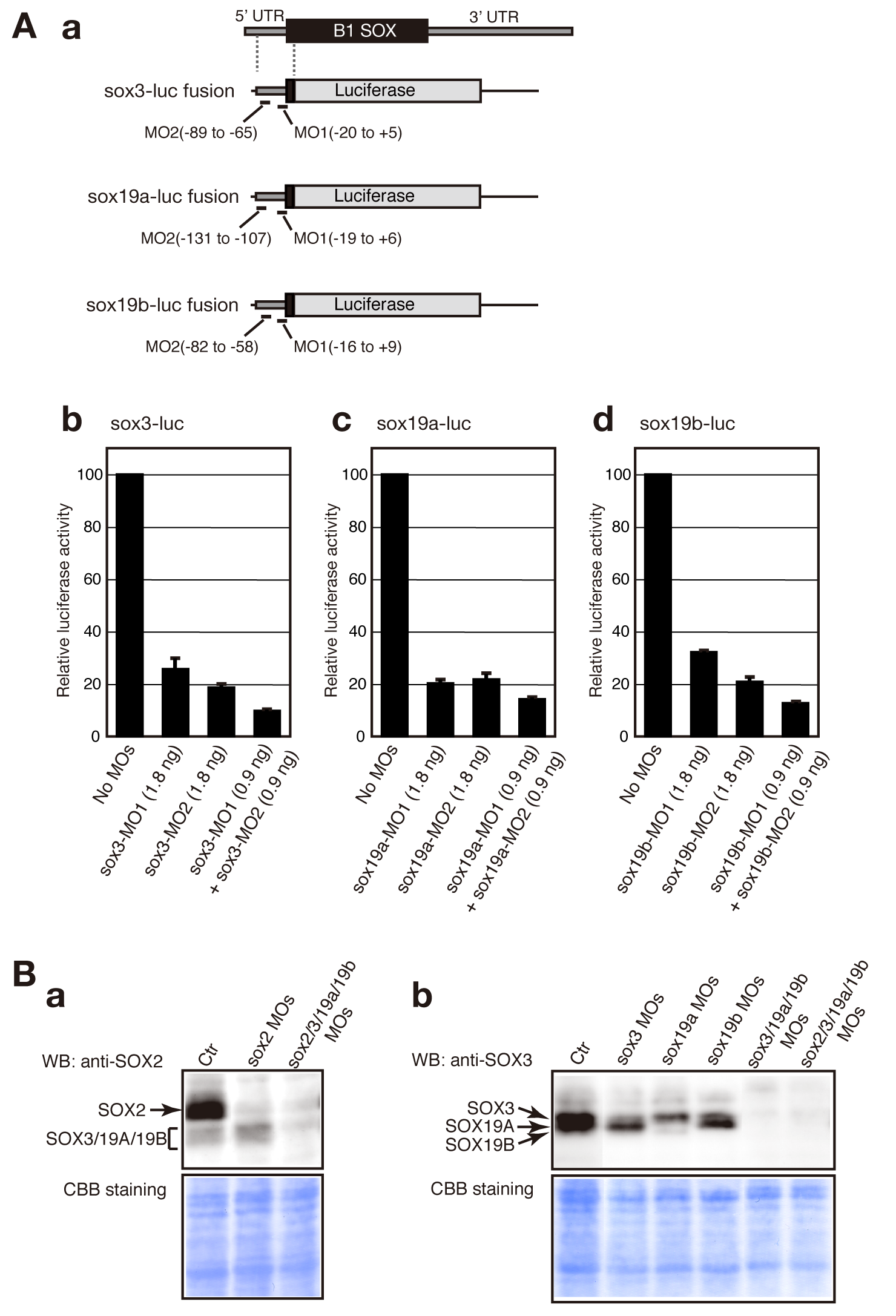

Supplement: Figure S2 — Evaluation of the knockdown efficiency of MOs targeting sox2, sox3, sox19a and sox19b. (A) Quantitative evaluation of the knockdown efficiency of MOs targeting sox3, sox19a and sox19b using a luciferase assay. (a) Schematic representation of sox3-luc, sox19a-luc and sox19b-luc fusion RNA constructs. (b–d) Inhibition levels caused by the injection of sox3-MOs (b), sox19a-MOs (c) and sox19b-MOs (d) were quantitatively measured using the luciferase assay-based system. Luciferase activity was measured using more than 20 injected embryos of the tailbud to early somite stages per sample. The luciferase activity generated by each sox-luc fusion in the absence of MOs was arbitrarily assigned a value of 100. Data are shown as the average values of three independent injection experiments with standard errors. (B) Inhibition of endogenous B1 SOX expression analyzed by western blotting. Lysates for SDS-PAGE were prepared using tailbud to early somite stage embryos that had been injected with the indicated MOs. The knockdown conditions were the same as those described in Figure 1. A seven-embryo equivalent amount of the lysate was used per lane. Western blotting was performed using an anti-SOX2 antibody that weekly cross-reacts with SOX3/19A/19B (a) and an anti-SOX3 antibody that preferentially detects SOX3/19A/19B (b). Note that the SOX19B expression levels are low at these stages. (0.96 MB TIF) [file pgen.1000936.s002.tif]

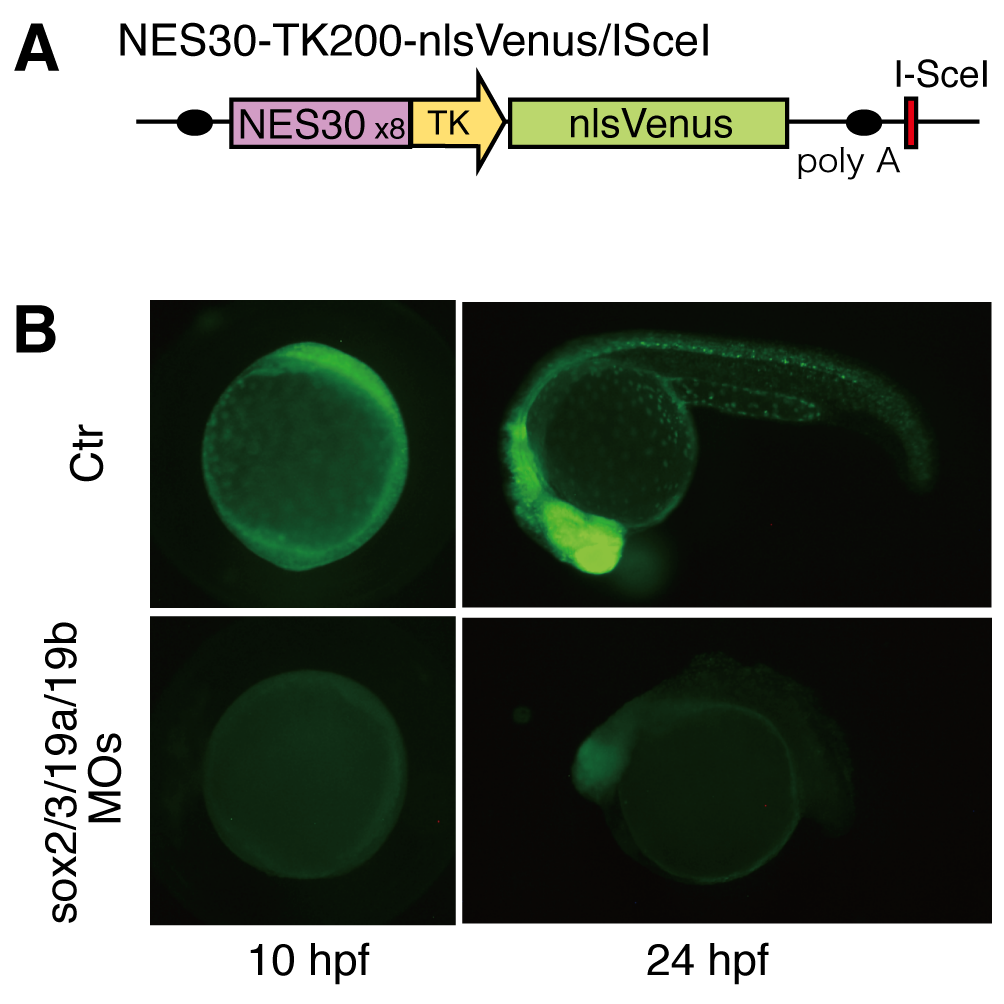

Supplement: Figure S3 — B1 sox activity is efficiently eliminated from the zebrafish embryo by B1 sox QKD. (A) Schematic representation of the NES30-TK200-nlsVenus/ISceI transgene construct used. NES30 is the 30-bp nestin enhancer core sequence, which is composed of SOX and POU binding sites. (B) NES30-driven nlsVenus expression (controls in upper panels) was abolished by injection of the MOs for QKD (lower panels), confirming effective depletion of B1 SOX activity from the embryo. (0.67 MB TIF) [file pgen.1000936.s003.tif]

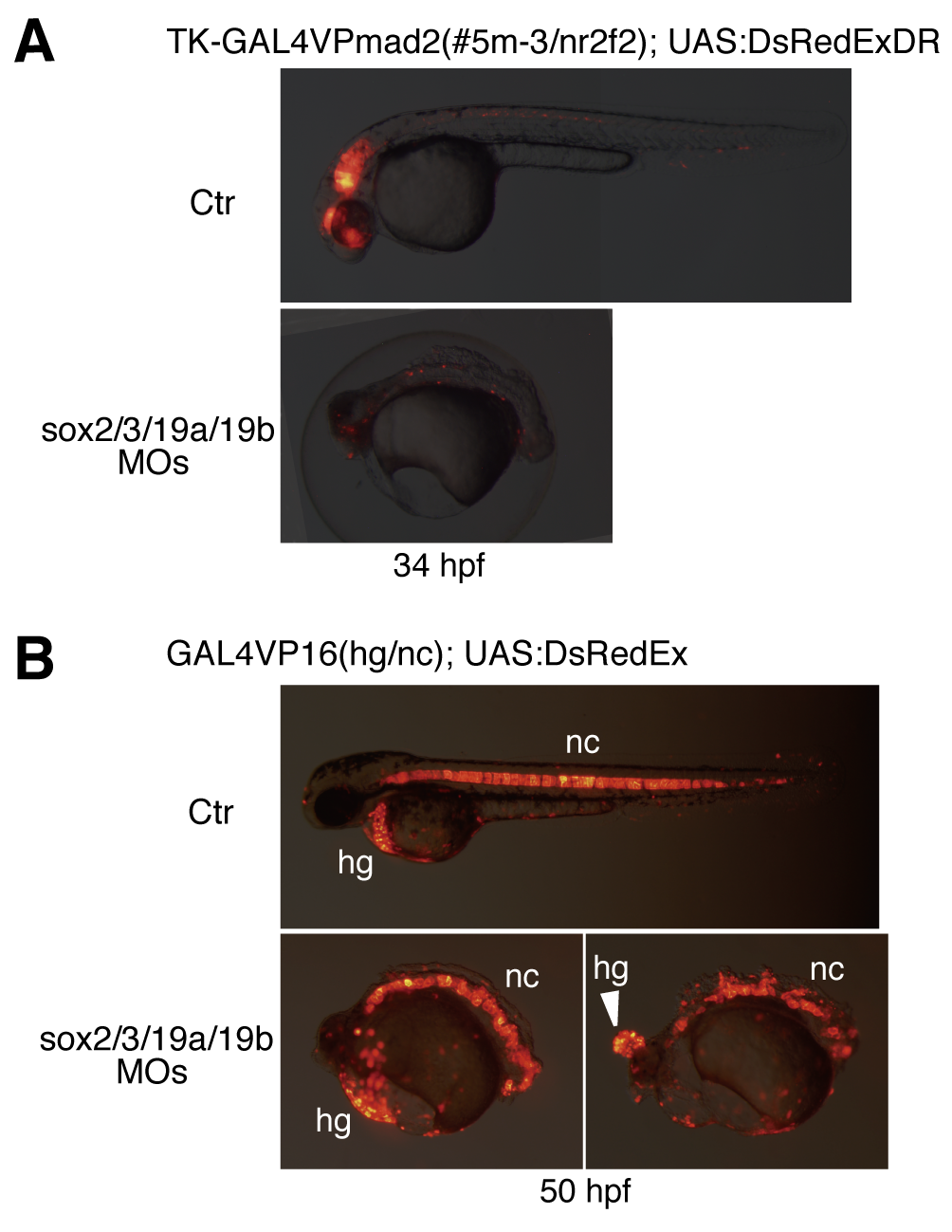

Supplement: Figure S4 — Effects of the B1 sox QKD revealed using transgenic lines with the GAL4-medeated reporter expression in the CNS and axial mesoderm. (A) Effects of QKD on CNS development were examined using the GAL4 enhancer trap line, which reports the activity of nr2f2 (couptfβ) neural enhancers. Double transgenic embryos harboring GAL4VPmad2 (#5m-3) and UAS:DsRedExDR normally show reporter expression in the CNS, which mimics neural nr2f2 expression. Injection of the MOs for QKD abolished this reporter expression, indicating impairment of CNS development. (B) Effects of QKD on axial mesoderm development, examined using the GAL4VP16(hg/nc) line. Double transgenic embryos harboring the GAL4VP16(hg/nc) transgene and UAS:DsRedEx show reporter expression in the hatching gland (hg) and notochord (nc). Strong expression of UAS:DsRedEx was observed after 1 dpf in this line. Injection of the MOs for QKD did not reduce reporter expression, indicating normal axial mesoderm differentiation in the morphants. However, hatching gland cells remained as a single ball-like structure in the heads of the severe morphants (arrowhead). (1.12 MB TIF) [file pgen.1000936.s004.tif]

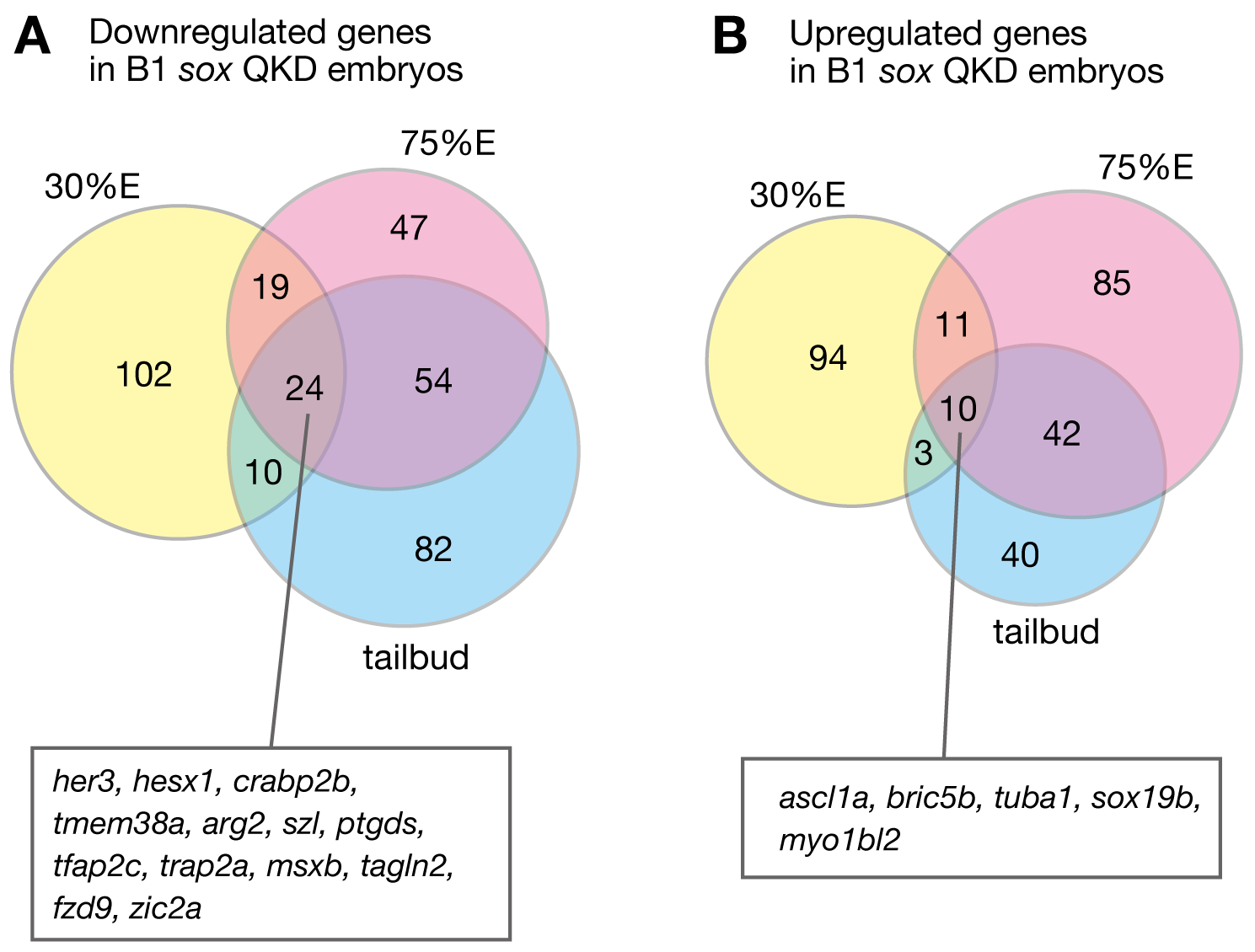

Supplement: Figure S5 — Gene expression profiles of the QKD embryos analyzed by microarray. Venn diagrams of genes that were found to be downregulated (A) and upregulated (B) in the B1 sox QKD embryos. Microarray analysis was carried out to compare gene expression profiles at the 30%E, 75%E, and tailbud stages between wild-type embryos and the QKD embryos. The numbers of Affymetrix zebrafish microarray probes that showed more than a twofold decrease (Table S2) or increase (Table S3) in the QKD embryos are shown. Annotated genes that were altered in all three stages are listed on the bottom in order of fold change at 75%E. (0.42 MB TIF) [file pgen.1000936.s005.tif]

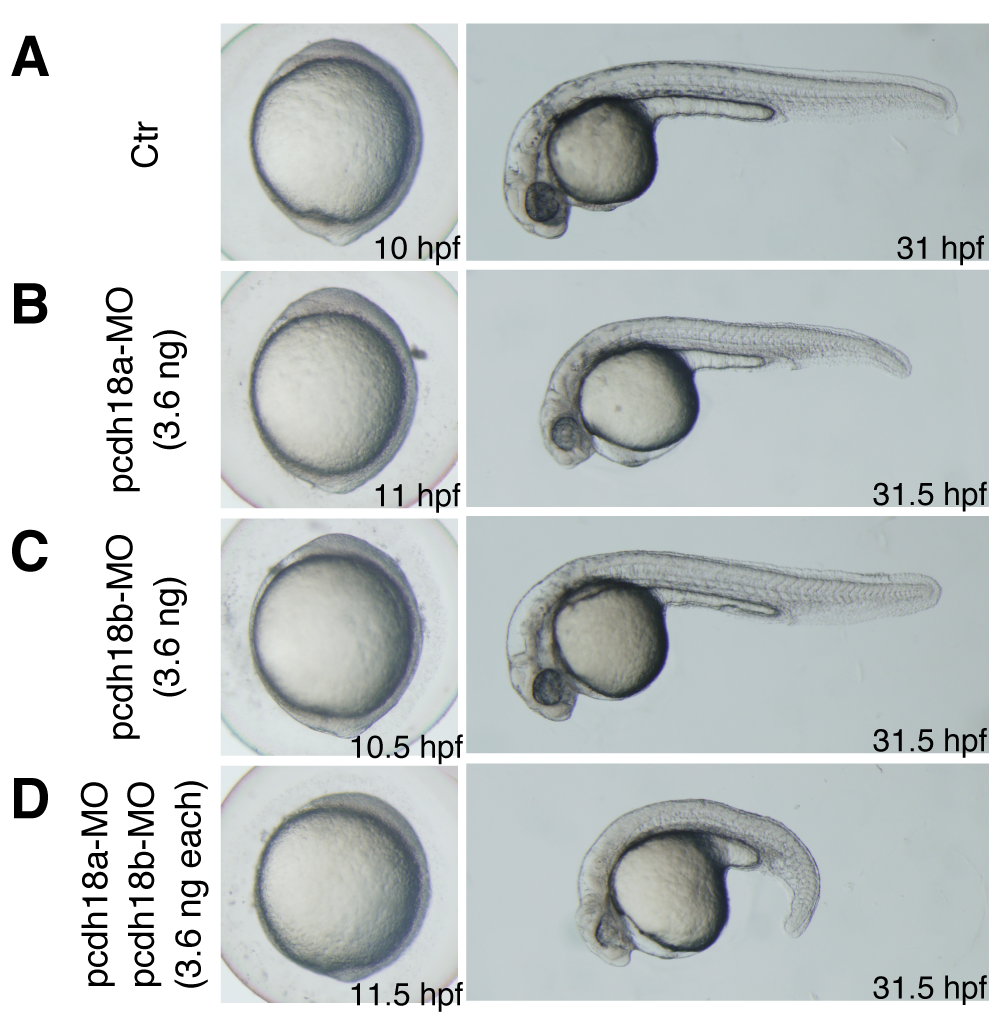

Supplement: Figure S6 — Phenotypes of pcdh18a and pcdh18b knockdowns. Bright-field images of live embryos at 10–11.5 hpf and 31–31.5 hpf are shown. (A) Uninjected control (Ctr) embryos. (B–D) Single and double knockdowns of pcdh18a/18b. Moderate amounts of MO (3.6 ng) were used for single KD (B,C) and a 1∶1 mixture of two MOs (3.6 ng each) was injected for double KD (D). Double knockdown embryos showed a shorter anteroposterior axis than the single knockdown embryos, indicative of more severe defects in C&E movements and suggesting a degree of functional redundancy between pcdh18a and pcdh18b. (1.83 MB TIF) [file pgen.1000936.s006.tif]

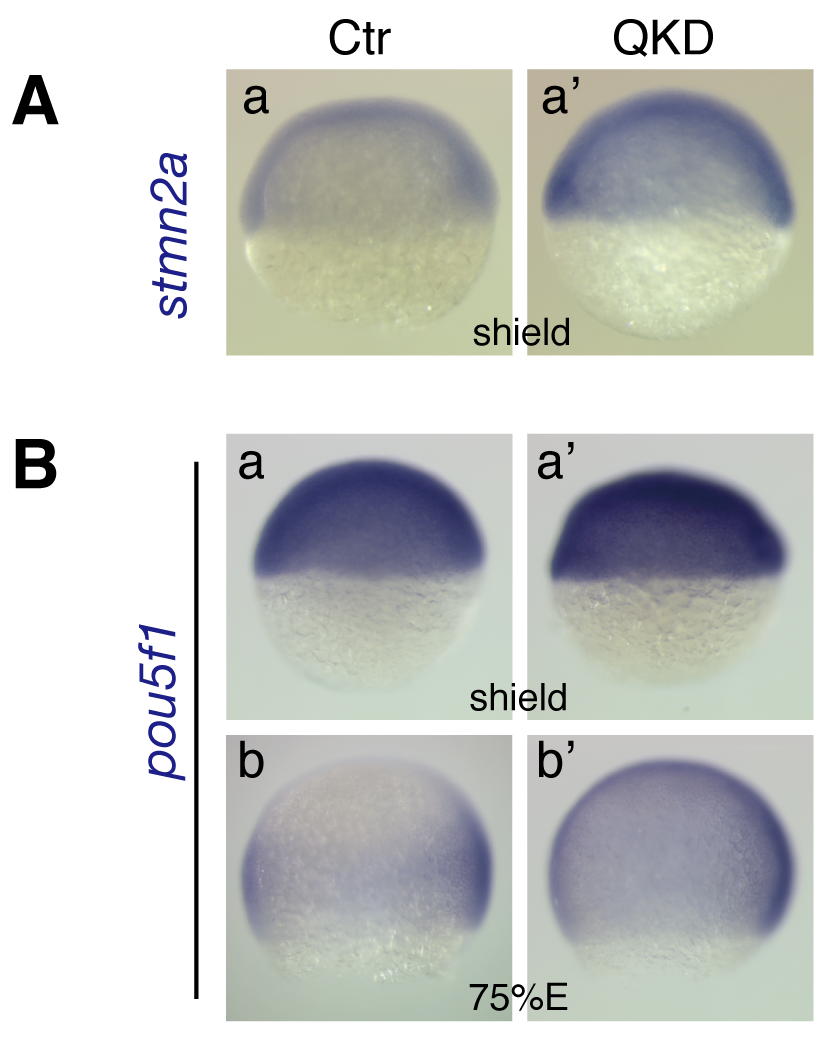

Supplement: Figure S7 — Expression of stmn2a and pou5f1 in the QKD embryos. (A) Expression of the stmn2a gene is upregulated in the QKD embryos at the shield stage. (B) Expression levels of pou5f1 were not affected in the B1 sox QKD embryos (our microarray data), whereas its expression domain was ventrally expanded at 75%E. (1.11 MB TIF) [file pgen.1000936.s007.tif]
