# Supplementary material for: B1 SOX Coordinate Cell Specification with Patterning and Morphogenesis in the Early Zebrafish Embryo
Source: PLoS Genet. 2010 May 6;6(5):e1000936. doi: 10.1371/journal.pgen.1000936 (PMC2865518; doi:10.1371/journal.pgen.1000936)
Supplement: Table S1 — Summary of gene expression analysis using in situ hybridization and/or RT-PCR. (0.39 MB DOC) [file pgen.1000936.s008.doc]

Table S1. Summary of gene expression analysis using in situ hybridization and/or RT-PCR

| Gene symbol | Gene name | Expression profile^a^ | Expression analysis in  B1 *sox* QKD embryos | |
| --- | --- | --- | --- | --- |
|  |  |  | in situ hybridization | RT-PCR |
| *ascl1a* | *achaete-scute complex-like 1a* | Not detectable until tailbud stage. Ventral diencephalon, presumptive epiphysis [1,2]. | Aberrantly upregulated (Fig. 5) | Aberrantly upregulated (Fig. 5) |
| *bmp2b*  *(swr)* | *bone morphogenetic protein 2b*  *(swirl)* | Blastoderm; ventral regions (animal and marginal parts) of late blastula and gastrula [3,4]. Absent in MZ*spg* mutant [5]. | Decreased (Fig. 4) | Decreased (Fig. 6, data not shown) |
| *bmp4* | *bone morphogenetic protein 4* | Blastoderm; ventral marginal zone of late blastula and gastrula [3,4]. Absent in MZ*spg* mutant [5]. | Slightly increased in blastula; decreased after shield stage (Fig. 4, data not shown) | Slightly increased in blastula; decreased after shield stage (data not shown) |
| *bmp7*  *(snh)* | *bone morphogenetic protein 7*  *(snailhouse)* | Blastoderm; ventral regions (animal and marginal parts) of late blastula and gastrula [6,7]. Absent in MZ*spg* mutant [5]. | Decreased (Fig. 4) | Decreased (Fig. 6, data not shown) |
| *cdh1*  *(e-cad,*  *hab)* | *cadherin 1, epithelial*  *(E-cadherin, half baked)* | Maternal; blastoderm; EVL layer; highest in specific portions of epiblast and hypoblast [8,9]. Gastrulation defects in *cdh1* mutant, *half baked* [9,10]. | Unchanged (data not shown) | Unchanged (Fig. 4) |
| *chd* | *chordin* | Shield region; axial mesoderm [11,12]. Ventral expansion in *bmp* mutants [11]. | Slight ventral expansion until shield stage; expression domain was reduced after shield stage (Fig. 4) | Decreased after shield stage (Fig. 6, data not shown) |
| *cyp26a1* | *cytochrome P450, subfamily XXVIA, polypeptide 1* | Anterior neuroectoderm and involuting margin [13-15]. Zebrafish promoter sequence (2.5 kb) can drive reporter expression similar to that of *cyp26a1* [16]. | Neuroectoderm expression is lost (Fig. 5). | Decreased (data not shown) |
| *dlx3b* | *distal-less homeobox gene 3b* | Non-neural ectoderm during epiboly; edge of anterior neural plate. Reduced in *bmp* mutants [17]. Widened expression in C&E mutants [18]. | Decreased during epiboly (Fig. 4).  Neural plate edge expression is lost (Fig. 2). | Decreased (data not shown) |
| *eng2a* | *engrailed 2a* | Midbrain-hindbrain boundary [19]. | Anterolaterally shifted (Fig. 3) | NA^b^ |
| *eve1* | *even-skipped-*  *like 1* | Ventral and lateral regions of marginal zone [20]. Absent in LiCl-treated dorsalized embryos [20]. | Decreased (Fig. 4) | Decreased (Fig. 4) |
| *foxd3*  *(fkd6)* | *forkhead box D3* | Dorsal involuting mesoderm; neural crest; tailbud [21]. Neural crest expression is reduced in *mdkb* knockdown embryos [22]. | Neural crest expression is Decreased (Fig. 5) | NA |
| *foxi1* | *forkhead box I1* | Non-neural ectoderm; presumptive otic placode [23]. | Decreased during epiboly (Fig. 3) | Decreased during epiboly (data not shown) |
| *gbx1* | *gastrulation brain homeobox 1* | Caudal part of neuroectoderm (hindbrain primordium); midbrain-hindbrain boundary, rhombomere 4, spinal cord [24]. | Anterolaterally shifted (Fig. 3) | NA |
| *gata2a* | *GATA-binding protein 2a* | Presumptive ectoderm; non-neural ectoderm [2,25]. Reduced or absent in *bmp* mutants [17]. Directly activated by Bmp signaling [26]. | Decreased (Fig. 4) | Decreased (Fig. 4) |
| *gsc* | *goosecoid* | Dorsal margin; hypoblastic part of shield; prechordal plate mesoderm [27]. | Slight ventral expansion (Fig. 4). | Slightly upregulated at sphere stage; unchanged at later stages (data not shown). |
| *her3* | *hairy-related 3* | Dorsal region of the epiblast; inter-proneuronal domains [28,29]. 4.7 kb promoter fragment mostly recapitulates *her3* expression pattern (Hans et al., 2004). | Completely lost (Fig. 5) | Completely lost (Fig. 5) |
| *hesx1*  *(anf)* | *homeo box expressed in ES cells 1*  *(anterior neural folds homolog)* | Anterior neuroectoderm; anterior-most neuroectoderm [30]. Chicken promoter sequence drives reporter expression in zebrafish embryos [31]. Forebrain defects in mouse knockout [32]. | Completely lost (Figs. 2, 3) | Completely lost (Fig. 5) |
| *hgg1*  *(ctsl1b)* | *hatching gland 1 (cathepsin L, 1b)* | Anterior prechordal plate mesoderm; presumptive hatching grand cells [27]. | Mislocalized (Fig. 2) | NA |
| *hoxb1a* | *homeo box B1a* | Posterior neuroectoderm (hindbrain, spinal cord); strong in rhombomere 4 [33]. | r4 expression is expanded (Fig. 5). | NA |
| *hoxb1b* | *homeo box B1b* | Posterior neuroectoderm (hindbrain, spinal cord) [34]. | Anterolaterally shifted (Figs. 2, 3) | NA |
| *krox20*  *(egr2b)* | *krox20*  *(early growth response 2b)* | Rhombomere 3/5 and r5-derived neural crest cells [35]. Reduced or absent in Cyp26-depleted embryos [15]. | Lost except for neural crest expression (Fig. 3) | Absent (data not shown) |
| *mafba*  *(val, kr)* | *v-maf musculoaponeurotic fibrosarcoma oncogene family, protein Ba (valentino, kreisler)* | Rhombomere 5/6 [36]. Reduced in *cyp26a1* mutant [14]. | Expression domain is narrowed with expression levels reduced (Fig. 5). | NA |
| *mdkb* | *midkine-related growth factor b* | Epiblast; neuroectoderm [22]. | Completely lost (Fig. 5) | NA |
| *myod1* | *myogenic differentiation 1* | Adaxial cells; somite [37]. Widened in C&E mutants [38]. | Widened (Fig. 3) | NA |
| *ndr2*  *(cyc)* | *nodal-related 2 (cyclops)* | Blastoderm margin; hypoblast layer of shield; prechordal plate and anterior notochord [39]. | Unchanged (Fig. 5) | Slightly upregulated at sphere stage; unchanged at later stages (data not shown). |
| *neurog1*  *(ngn1)* | *neurogenin 1* | Primary neurons; proneuronal domains [28,40,41]. Phylogenetically conserved sequences (LSE, ANPE, LATE) are required for its neuronal expression [42]. | Almost completely lost (Fig. 2) | Almost completely lost (Fig. 5) |
| *nkx1.2la*  *(sax1)* | *NK1 transcription factor related 2-like a* | Posteior neuroectoderm [43]. | Almost completely lost (Fig. 3) | NA |
| *nog1* | *noggin 1* | Dorsal margin; anterior hypoblastic part of shield, prechordal plate and axial mesoderm [44]. | Slight ventral expansion until shield stage (Fig. 3) | Unchanged (data not shown) |
| *ntl* | *no tail*  *(brachyury)* | Notochord [45]. Shorter and wider expression in *wnt11* mutant [46] and in *wnt5b/11* double mutant [18]. | Widened (Figs. 2, 3) | Unchanged (data not shown) |
| *oep*  *(tdgf1, cripto)* | *one-eyed pinhead (teratocarcinoma-derived growth factor 1, cripto)* | Maternal; blastoderm; margin; axial and paraxial hypoblast, neuroectoderm [47]. | Neuroectoderm expression is specifically lost (Fig. 5). | NA |
| *otx2* | *orthodenticle homolog 2* | Anterior neuroectoderm[48]. Ventral expansion in *bmp* mutants [17]. | Expression domain is expanded (Fig. 3). | Unchanged (data not shown) |
| *pax2a*  *(noi)* | *paired box*  *gene 2a*  *(no isthmus)* | Midbrain-hindbrain boundary [49]. Lateral and/or ventral expansion in *bmp* mutants [50]. | Anterolaterally shifted (Fig. 2) | NA |
| *pou5f1*  *(spg,*  *pou2)* | *POU domain, class 5, transcription factor 1*  *(spiel ohne grenzen)* | Maternal; blastoderm; neuroectoderm; mid-hindbrain boundary [51,52]. | Early expression is unchanged. Neuroectoderm expression was ventrally expanded (Fig. S7). | NA |
| *pcdh18a* | *protocadherin 18a* | Epiblast, neuroectoderm [53]. | NA | Decreased (Fig. 4) |
| *pcdh18b* | *protocadherin 18b* | Epiblast, neuroectoderm [54]. | NA | Decreased (Fig. 4) |
| *rest*  *(nrsf)* | *RE1-silencing transcription factor (nrsf)* | Not reported for zebrafish. Zebrafish cDNA [55]. Mouse knockout [56]. | NA | Unchanged (Fig. 5) |
| *rx3*  *(eym, chk)* | *retinal homeobox gene 3*  *(eyes missing, chokh)* | Eye field, hypothalamus [57]. *rx3* mutnat lacks eyes [58]. | Almost completely lost (Fig. 3) | Almost completely lost (Fig. 5) |
| *shha*  *(syu)* | *sonic hedgehog a*  *(sonic-you)* | Shield; notochord; floor plate, ventral floor of brain (after gastrulation) [59,60]. | Neural expression is lost (Fig. 5). | NA |
| *shhb*  *(twhh)* | *sonic hedgehog b*  *(tiggy winkle hedgehog)* | Shield; floor plate; ventral floor of brain [59,61]. Prechordal plate expression reported in cavefish [62]. | Neural expression is lost (Fig. 5). | NA |
| *sox1a* | *SRY-box containing gene 1a* | Forebrain, hindbrain [63]. | NA | Unchanged until tailbud stage; decreased after early somite stages (Fig. S1) |
| *sox1b* | *SRY-box containing gene 1b* | Forebrain [63]. | NA | Initially increased; decreased after early somite stages (Fig. S1) |
| *sox2* | *SRY-box containing gene 2* | Presumptive ectoderm; neuroectoderm (strong in anterior neuroectoderm) [63]. | Ventrally expanded (Fig. 3) | Increased (Fig. S1) |
| *sox3* | *SRY-box containing gene 3* | Blastoderm; presumptive ectoderm; neuroectoderm [13,63]. | Ventrally expanded (Fig. 3) | Increased (Fig. S1) |
| *sox19a* | *SRY-box containing gene 19a* | Blastoderm; presumptive ectoderm; neuroectoderm [63,64]. | Ventrally expanded (Fig. 3) | Increased (Fig. S1) |
| *sox19b*  *(sox31)* | *SRY-box containing gene 19b (31)* | Maternal; blastoderm; presumptive ectoderm; neuroectoderm [63,65]. | Ventrally expanded (Fig. 3) | Increased (Fig. S1) |
| *stmn2a*  *(scg10b)* | *stathmin-like 2a*  *(superior cervical ganglia 10b)* | Neurons of the CNS and ganglions [2,66]. No aberrant expression in *Rest* knockout mice [56]. | Aberrantly upregulated (Fig. S7) | Aberrantly upregulated (Fig. 5) |
| *szl*  *(ogo, mes)* | *sizzled*  *(ogon, mercedes)* | Ventral regions of blastula and gastulura. Dependent on Bmp signaling [67]. | Decreased (Fig. 4) | Decreased (Fig. 4) |
| *tuba1* | *tubulin, alpha 1* | Brain, spinal chord [68]. | NA | Increased after shield stage (Fig. 5) |
| *wnt4a* | *wingless-type MMTV integration site family, member 4a* | Forebrain, hindbrain, spinal cord, anterior lateral plate mesoderm (after tailbud stage) [69,70]. | NA | Decreased after tailbud stage (Fig. 4) |
| *wnt5b*  *(ppt)* | *wingless-type MMTV integration site family, member 5b (pipetail)* | Maternal; germ ring; posterior paraxial mesoendoderm [18]. | NA | Decreased during early epiboly stages (Fig. 4) |
| *wnt11*  *(slb)* | *wingless-type MMTV integration site family, member 11 (silberblick)* | Germ ring; paraxial head mesoderm; neuroectoderm [46] | Decreased during early epiboly stages (data not shown) | Decreased during early epiboly stages (Fig. 4) |
| *wnt11r* | *wingless-type MMTV integration site family, member 11 related* | Forebrain, spinal chord (after tailbud stage) [69,70]. | NA | Decreased after tailbud stage (Fig. 4) |
| *zic1*  *(opl)* | *zic family member 1 (*odd-paired  -like*)* | Presumptive forebrain, weak expression in lateral edges of neural plate [71]. Knockdown of *zic1* causes forebrain midline defects [72]. | Almost completely lost (Fig. 3) | Almost completely lost (Fig. 5) |
| *zic2b* | *zic family member 2b* | Blastoderm; presumptive ectoderm; neuroectoderm [73]. | Expression domain is ventrally expanded (Fig. 3). | Unchanged (data not shown). |

^a^ Gene expression profiles in wild-type and mutant/morphant zebrafish embryos (until early somite stages). Notes on mutant/morphant phenotypes and the regulatory sequences that were analyzed in our ChIP analysis.

^b^ Not assessed.

**References for Table S1**
