# Supplementary material for: B1 SOX Coordinate Cell Specification with Patterning and Morphogenesis in the Early Zebrafish Embryo
Source: PLoS Genet. 2010 May 6;6(5):e1000936. doi: 10.1371/journal.pgen.1000936 (PMC2865518; doi:10.1371/journal.pgen.1000936)
Supplement: Table S4 — Morpholino antisense oligonucleotides. (0.11 MB DOC) [file pgen.1000936.s011.doc]

Table S4. Morpholino antisense oligonucleotides

| MO name | MO sequence | Reference |
| --- | --- | --- |
| sox2-MO2 | GAGAGGCTGCTGAAGTTACCTTAGC | Kamachi et al., 2008 [20] |
| sox2-MO2(5mis)^a^ | GAcAGcCTGCTcAAGTTACgTTAcC | This study |
| sox2-MO3 | GAAAGTCTACCCCACCAGCCGTAAA | Kamachi et al., 2008 [20] |
| sox2-MO3(5mis)^a^ | GAAAcTCTAgCCgACgAGCCcTAAA | This study |
| sox3-MO1 | TACATTCTTAAAAGTGGTGCCAAGC | This study |
| sox3-MO1(5mis)^a^ | TAgATTgTTAAAAGTGcTGCgAAcC | This study |
| sox3-MO2 | GAAGTCAGTCAAAAGTTCAGAGAGC | This study |
| sox3-MO2(5mis)^a^ | GAAcTCAcTCAAAAcTTgAGAcAGC | This study |
| sox19a-MO1 | GTACATGGCTGCCAACAGAAGTTAG | This study |
| sox19a-MO1(5mis)^a^ | GTAgATcGCTGCgAACAcAAcTTAG | This study |
| sox19a-MO2 | AAAACGAGAGCGAGCCGTCTGTAAC | This study |
| sox19a-MO2(5mis)^a^ | AAAAgGAcAGCGAcCCcTCTcTAAC | This study |
| sox19b-MO1 | GTACATCATGCCACTTCTCGCTTTG | This study |
| sox19b-MO1(5mis)^a^ | GTAgATgATGCgACTTgTCGgTTTG | This study |
| sox19b-MO2 | ACGAGCGAGCCTAATCAGGTCAAAC | This study |
| sox19b-MO2(5mis)^a^ | ACcAGCcAGCgTAATCAaGTgAAAC | This study |
| pcdh18a-MO(UTR) | TCCGTCAGGCACTGCAAAAATATAC | Aamar et al., 2008 [41] |
| pcdh18b-MO1 | ATTGTTCGGTGTAGATTAAAGCATG | This study |
| p53-MO | GCGCCATTGCTTTGCAAGAATTG | Robu et al., 2007 [30] |

^a^ Negative control MOs were designed to contain 5-base mismatches (indicate by lowercase letters).

Table S5. Primers and conditions for RT-PCR

| Gene | Forward | Reverse | Product size (bp) | PCR cycle | Annea-ling temp. | Reference |
| --- | --- | --- | --- | --- | --- | --- |
| *ascl1a* | GCTCCTGGACTTCACCAACTGG | TGGATTCCCGCTCTTCCCTC | 214 | x30 | 61 | This study |
| *bactin1* | CTGGGTATGGAATCTTGCGGTATC | CGAGAGTTTAGGTTGGTCGTTCG | 397 | x23 | 57 | {Okuda, 2006 #2} |
| *bmp2b* | ACCTACAGCCATGACGGTCAAG | TGGGGAGATTGTTCTCATCGG | 428 | x26 | 57 | This study |
| *bmp4* | GCTGTATGTGGATTTCAGCGACG | GGATGTTTTTGTGGTAAGAGTCTCCG | 319 | x27 | 57 | This study |
| *bmp7* | AAGCATCAGGAATCCACCTCCG | AGTTTGGGTCCAGCAGCGAGTC | 578 | x27 | 57 | This study |
| *cdh1* | TCCTCCCTACGACTCTCTGCTG | ACATCTTTCCACTGAGCCATCG | 451 | x26 | 57 | This study |
| *chd* | TGAAGACCAATGCTGTCCCATC | CATCATCTCCTCGTCCTCCAGAG | 331 | x25 | 57 | This study |
| *dlx3b* | CCATCACGAATACTACCAGAGCCC | TTCCGTTTACCATCCGCACTTC | 224 | x27 | 57 | This study |
| *eve1* | CCTTTCATAAACGAACTTGGCTTG | CTTCCTCTCCTTGGTCACTGTCAG | 227 | x27 | 59 | This study |
| *foxi1* | CAAATCCTCTCCCTCTCCGTCC | AAGCCACTGTTGTTGTGCGATG | 237 | x29 | 59 | This study |
| *gata2* | TCCTTCTTCTCCGACATCCACC | GCTCCACAGTTCACACATTCACG | 318 | x29 | 57 | This study |
| *her3* | CACTGATGGAGAAAAAAAGAAGGG | CCGACGCTCGTAGATAATGACTTAC | 255 | x27 | 57 | This study |
| *hesx1* | TCGACAGCATCCTGGGACTG | CTTGATCTGAACACTGGAGAAGGC | 242 | x26 | 57 | This study |
| *neurog1* | CATCTCCCAGCCCACCAATAAG | GCTTTGTGTCGTCAGGAAACGC | 348 | x30 | 57 | This study |
| *nog1* | GTGGCTGTGGTCCTACTCGTTC | GCACTCTGTGATGATGGGATACTG | 247 | x28 | 57 | This study |
| *pcdh18a* | CAGATTTCATTTTTGGTCCAGGC | ACAACAGAGCAGGAGAGGTCACC | 242 | x30 | 59 | This study |
| *pcdh18b* | CCCCACTGCTAAGTAACCACACAG | GCTTTCTGGATAGTCCCGTAGGC | 317 | x28 | 59 | This study |
| *rest* | GCAGCCAGTGCTCATACTTTTCTG | GAGTGGTTTGGGTCCGTTGTG | 251 | x30 | 59 | This study |
| *rx3* | CGGGAAAGACAGGCAAAGACAC | TACTCGGACTTCGGGCAGGTTG | 290 | x27 | 59 | This study |
| *sox1a* | ACCCTGCTTAAAAAGGACAAGTACTCG | GGGGACTTATATTGGGTTCGGAC | 493 | x30 | 57 | {Okuda, 2006 #2} |
| *sox1b* | CAGCCCCCTCTCAAACTCTCAG | TGAAGCACCCACTCTTATTCGC | 591 | x30 | 57 | {Okuda, 2006 #2} |
| *sox2* | CGCTCCAGTACAACTCCATGACC | TTACATATGCGATAAGGGAATCGTGC | 347 | x25 | 57 | {Okuda, 2006 #2} |
| *sox3* | GGTGCCAACGCTGTCAACAAC | TCTCTCAGGTCTCCCAAACAAGC | 377 | x23 | 57 | {Okuda, 2006 #2} |
| *sox19a* | ATCTGTCCGCCTTGCAGTATCCA | TTTCCTGATCAGATGTGTGTGAGAGG | 328 | x25 | 57 | {Okuda, 2006 #2} |
| *sox19b* | GATCTGTCAGCTCTGCAGTACCCG | TCAGATGTGAGTGAGGGGAACAGTTC | 309 | x27 | 57 | {Okuda, 2006 #2} |
| *stmn2a* | CCACAGACACGCAACAACATTG | ATGGCTTTGAGCAGCACATCCC | 287 | x30 | 61 | This study |
| *szl* | CTCGCTCTGCCTCAACGACTTC | CTTTCTTCAGCCACGGGAACAG | 284 | x29 | 59 | This study |
| *tuba1* | GGTACGTGGGTGAGGGTATGG | TCGCAAGGATTGACCTTTTAGC | 226 | x28 | 59 | This study |
| *wnt4a* | GACTCATCCAGAGGCAGGTTCAG | ACGGTCACATCCACACTTGTCC | 278 | x30 | 59 | This study |
| *wnt5b* | GCCTATCTCAGGGTCAGAGGAAG | CGACTCACAGCATTCACAACACC | 235 | x27 | 59 | This study |
| *wnt11* | CAAGACATCAGCACCATCTCCG | TCCACCAGCACCTCTGTGTAGG | 299 | x26 | 57 | This study |
| *wnt11r* | ACTGGACCTAAAGGACATCGCC | CTCCGTGTAAGGGTTGTAGCCTC | 289 | x30 | 57 | This study |
| *zic1* | ATTTGCTTTTGGGAAGAGTGCG | CGGAGAGAACTGGGATGTGTGTAG | 326 | x27 | 59 | This study |

Table S6. Primers and conditions for ChIP-PCR

| Target | Forward | Reverse | Product size (bp) | PCR cycle | Annea-ling temp. | Reference |
| --- | --- | --- | --- | --- | --- | --- |
| *bactin2* | AACACAACACAGGATCATGGAG | CATTGCTACACTTGCTTCTTGC | 244 | x30 | 57 | {Wardle, 2006 #40} |
| *cyp26a1* | CTCAGGATTGTCTGCCTTCTACAG | AGGTCTCCTCCTGTTAACTTCCTC | 167 | x30 | 57 | This study |
| *her3* (proximal) | AGAGAGCAACCTGAAGCTGATTGG | GCTGCAGCCATTGTCCTTAAATGC | 217 | x30 | 57 | This study |
| *her3* (distal) | GCAGCAGGGCATTGTCATGTTGAA | TCAGTTCCGCTATTGTCAAGCA | 182 | x30 | 57 | This study |
| *hesx1* | CTGCTTCCAGCAAATTGCCAAG | TGAGGAAGCAGTCAACTAGTGGC | 193 | x30 | 57 | This study |
| *neurog1* (LSE) | AAAAGCCCTGTTCCTGAGCTCC | GAAGTCTGAGCTTGACTCGACAAAG | 254 | x30 | 57 | This study |
| *pcdh18a* | AATGAGACAGACGAGGCGACAGAT | CAAACAATGACCACAGCAGGTGGA | 157 | x30 | 57 | This study |
