# Supplementary material for: B1 SOX Coordinate Cell Specification with Patterning and Morphogenesis in the Early Zebrafish Embryo
Source: PLoS Genet. 2010 May 6;6(5):e1000936. doi: 10.1371/journal.pgen.1000936 (PMC2865518; doi:10.1371/journal.pgen.1000936)
Supplement: Table S6 — Primers and conditions for ChIP-PCR. (0.04 MB DOC) [file pgen.1000936.s013.doc]

Table S6. Primers and conditions for ChIP-PCR

| Target | Forward | Reverse | Product size (bp) | PCR cycle | Anneal-ing temp. | Reference |
| --- | --- | --- | --- | --- | --- | --- |
| *bactin2* | AACACAACACAGGATCATGGAG | CATTGCTACACTTGCTTCTTGC | 244 | x30 | 57 | Wardle et al., 2006 [67] |
| *cyp26a1* | CTCAGGATTGTCTGCCTTCTACAG | AGGTCTCCTCCTGTTAACTTCCTC | 167 | x30 | 57 | This study |
| *her3* (proximal) | AGAGAGCAACCTGAAGCTGATTGG | GCTGCAGCCATTGTCCTTAAATGC | 217 | x30 | 57 | This study |
| *her3* (distal) | GCAGCAGGGCATTGTCATGTTGAA | TCAGTTCCGCTATTGTCAAGCA | 182 | x30 | 57 | This study |
| *hesx1* | CTGCTTCCAGCAAATTGCCAAG | TGAGGAAGCAGTCAACTAGTGGC | 193 | x30 | 57 | This study |
| *neurog1* (LSE) | AAAAGCCCTGTTCCTGAGCTCC | GAAGTCTGAGCTTGACTCGACAAAG | 254 | x30 | 57 | This study |
| *pcdh18a* | AATGAGACAGACGAGGCGACAGAT | CAAACAATGACCACAGCAGGTGGA | 157 | x30 | 57 | This study |
